# Printed sustainable elastomeric conductor for soft electronics

**DOI:** 10.1038/s41467-023-42838-7

**Published:** 2023-11-06

**Authors:** Jian Lv, Gurunathan Thangavel, Yangyang Xin, Dace Gao, Wei Church Poh, Shaohua Chen, Pooi See Lee

**Affiliations:** 1 School of Materials Science and Engineering, Nanyang Technological University, 50, Nanyang Avenue, Singapore, 639798 Singapore; 2 Singapore-HUJ Alliance for Research and Enterprise (SHARE), Smart Grippers for Soft Robotics (SGSR), Campus for Research Excellence and Technological Enterprise, Singapore, 138602 Singapore; 3 Present Address: Frontier Institute of Science and Technology, State Key Laboratory for Manufacturing Systems Engineering, Xi’an Jiaotong University, 710049 Xi’an, China; 4 Present Address: Advanced Materials Research Center, Technology Innovation Institute (TII), Masdar City, Abu Dhabi P.O. Box 9639, United Arab Emirates

**Keywords:** Electrical and electronic engineering, Electronic devices, Polymers

## Abstract

The widespread adoption of renewable and sustainable elastomers in stretchable electronics has been impeded by challenges in their fabrication and lacklustre performance. Here, we realize a printed sustainable stretchable conductor with superior electrical performance by synthesizing sustainable and recyclable vegetable oil polyurethane (VegPU) elastomeric binder and developing a solution sintering method for their composites with Ag flakes. The binder impedes the propagation of cracks through its porous network, while the solution sintering reaction reduces the resistance increment upon stretching, resulting in high stretchability (350%), superior conductivity (12833 S cm^−1^), and low hysteresis (0.333) after 100% cyclic stretching. The sustainable conductor was used to print durable and stretchable impedance sensors for non-obstructive detection of fruit maturity in food sensing technology. The combination of sustainable materials and strategies for realizing high-performance stretchable conductors provides a roadmap for the development of sustainable stretchable electronics.

## Abstract (web summary)

Printed stretchable electronics with sustainable elastomers are typically limited by their electro-mechanical performances. Herein, Lv et al. report a sustainable vegetable oil-based polyurethane-enabled printed conductor with high performance enabled by the porous binder and sintering of silver flakes.

## Introduction

Stretchable devices with the capability of consistent performance under mechanical strain have shown great potential in wearable health monitoring, human-machine interfaces, and soft robotics^1^. Among the various fabrication techniques, the printing of stretchable conductors has attracted massive attention owing to its relatively low cost, scalable fabrication, less material waste (additive manufacturing), and versatile material selection^2^. The incorporation of nano/micro conductive fillers with elastomeric binders has been intensively studied for the formulation of printable ink owing to their high conductivity and stability^3^. The conduction mechanism of the printed stretchable composites relies on the embedded percolation of conductive fillers, with elastomeric binders filling the interspace of the conductive networks. The binders for the printed stretchable inks can be silicone rubbers, fluorine rubbers, styrene elastomers, and polyurethane-based elastomers^3^. However, all of them are petro-based elastomers, causing concerns on sustainability and CO_2_ emissions for the wide application of printed stretchable devices. As sustainability is an important aspect of circular economy, the future development of deformable devices calls for bio-based feedstock, sustainable polymers or binders to replace petro-derived elastomers.

A serious impediment to the widespread use of sustainable printed electronics is the meager functional electro-mechanical performances. The mechanical property of the polymeric binders and interactions between binders and conductive fillers determine the stretchability and electrical performance of the conductor^4^. Under the mechanical deformation, the conductive fillers slide against each other while the elastic binders resist the cracking of the conductors and hold the conductive fillers together. A comparable or even better performance than traditional printed electrodes can stimulate the adoption of printed sustainable stretchable electrodes. However, the up-to-now trials for printed sustainable stretchable electronics are unsatisfactory. In the formulation of printed inks, the elastomeric binders account for a big amount in volume ratio, though with a low weight ratio as their densities (1–1.23) are much lower than that of metal conductor fillers. Natural polymers, including cellulose, chitosan, gelatin, and silk, have been extensively studied in the formulation of ink, but their non-extendable nature affects their application in soft and stretchable electronics^5^. Natural polymer-based composite hydrogels can be stretchable, but the conductivity is inferior compared with dry electrodes due to the existence of water in a high volume proportion. A sustainable printable conductor with high conductivity, stretchability, and low electrical hysteresis after cycling stretching is highly desirable. Alternative sustainable polymers and breakthrough strategies in endowing the electrode with high performance are required to make the present stretchable electronics more attractive and environmentally benign.

In this work, we report a sustainable vegetable oil-based polyurethane-enabled printed conductor (VegPU/Ag) delivering a high conductivity of 12,833 S cm^−1^, 350% stretchability, and low electrical hysteresis for up to 100% cycling stretching, realized by the co-existence of internal sintering among Ag flakes and porous VegPU binder. The well-designed sustainable and recyclable VegPU elastomeric binder is composed of bio-derived dynamic crosslinked covalent networks based on environmentally friendly polyols constructed from castor oil-based bifunctional ricinoleic acid (COBRA) and epoxidized soybean oil (ESBO). The key to attaining these unique characteristics is the dynamic relationship of triple interactive bonds in the VegPU binder, including reversible carbamate-oxime covalent (–NH–CO–O‧‧‧‧N=CRR’) bonds, carbamate–carbamate (–NH–COO‧‧‧‧H–N–) bonds, and hydrogen (–C=O‧‧‧‧H–N–) bonds within VegPU binder. After the printing, the uncured electrode was soaked into the green sintering solutions containing lactic acid and Cl^-^ to enable a room-temperature simultaneous binder curing and sintering of the Ag flakes. The curing enabled by the non-solvent-induced phase separation (NIPS) generated a porous structure of the binder while the sintering of Ag flakes was achieved simultaneously through the partial removal of the surfactant. The porous binder can reduce or impede the propagation of the cracking during the mechanical deformation, especially during the cyclic stretching; and the sintering reaction weakened the barrier for the electron transfer. Thus, the printed sustainable conductor can reach both high durabilities in mechanical deformation and high electrical conductivity. To show the application of our sustainable conductor, we fabricated stretchable impedance sensors mounted on the soft gripper that can realize the non-obstructive monitoring of the fruit maturity for smart fruit picking and sorting. The highly resilient conductor makes the sensor perform stably on the deformed soft gripper. Overall, the sustainable conductor with a high performance presented here provides a roadmap for the realization of sustainable stretchable electronics, which not only utilizes climate-friendly sustainable materials but also invents protocols in the fabrication of the sustainable conductor with comparable or even better performance than conventional stretchable conductors.

## Results

### Design of sustainable VegPU/Ag conductor

**Fig. 1:**
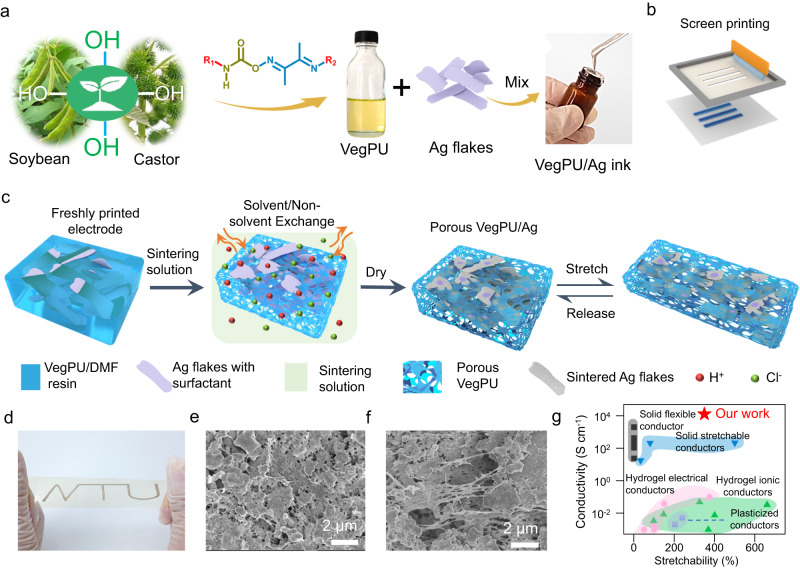
Design of sustainable VegPU/Ag conductor. **a** The formulation and **b** screen printing of the VegPU/Ag ink. **c** The schematics show the sintering solution treatment of freshly printed VegPU/Ag electrodes and subsequent stretching. **d** The photo image of one printed VegPU/Ag pattern on VegPU substrate with the presence of 100% strain. **e**, **f** The SEM images of the sintering solution-treated VegPU/Ag conductor at relaxed and stretched state (100% strain), respectively. **g** The performance comparison of VegPU/Ag with reported printable sustainable and biodegradable conductors, including solid flexible^17–21^, solid stretchable^22,23^, hydrogel electrical^24–28^, hydrogel ionic^29–33^ and plasticized conductors^34,35^.

Figure 1a, b show the formulation and printing of the VegPU/Ag ink. For the printed stretchable conductor, the stretchability comes from the elastomeric binders, while the electrical performance relies on the conductive fillers. As metal conductive fillers can be recollected, the sustainable binder plays a key role in enhancing the sustainability of printed stretchable electronics. Here, the sustainable VegPU binder made of sustainable polyols (SPOs) from COBRA and ESBO was developed using an environmentally friendly and low carbon footprint method, replacing the traditional fossil fuel-based polymer matrices. Among various plant oils, we chose castor oil and soybean oil as the most promising green polyols because of their distinctive long adaptable fatty acid structure that makes them the ideal soft segments (SS) for polyurethane preparation^6^. Ricinoleic acid (12 hydroxy-cis-9-octadecenoic acid) is the major hydroxylated aliphatic unsaturated fatty acid in castor oil (>90%) and a key substrate for the polymerization of precursors to produce sustainable VegPU^7,8^. Soybean oil is abundant and contains a high number of -C=C- double bonds, which allows for a wide range of potential modification routes^9–11^. The two vegetable oils contain dangling soft linear aliphatic chains that could play two key functions in VegPU binders: (i) forming van der Waals bonds by intertwining with each other to endow the VegPU with stretchability and reversibility, and (ii) disrupting the formation of significant carbamate hard segments (HS) to avoid VegPU binder crystallization. As a result of the abundance of sacrificial H-bonds and van der Waals forces, such a structure favors the formation of a reversible physically cross-linked dynamic network, resulting in highly stretchable and recyclable VegPU. This VegPU elastomer not only contributes to sustainable development, but also to addressing environmental issues, waste disposal, and the depletion of nonrenewable resources. Ag flake is one of the most promising conductive fillers for the printing of large-scale devices, for its ease of forming percolation, scalable fabrication, good stability, and high conductivity^3^. Meanwhile, compared with nano conductive fillers, micro-Ag flakes own the advantage of potential recollection during the recycling process^12^. Emerging liquid metals outperform the Ag flakes in terms of stretchability because of their flowing property, but they are liable to react with other metals when in contact with the integrated circuit and are hard to encapsulate due to their liquid state^13^.

The concepts of sustainable polymeric binders and substrates are widely used in coatings, plastics, and batteries, but their applications in printed stretchable conductors are obscure, due to the challenges of realizing both good conductivity and electromechanical properties. The traditional way of treating the freshly printed pattern is initiated by heat or UV light to remove solvent or crosslink the binder matrix, which normally generates dense matrices or binders that bind the conductive fillers. Most Ag flakes-based stretchable conductors are with a high loading of conductive fillers (Vol%>15) and thus defects inevitably exist on the polymeric binder matrix. Upon stretching, these defects are liable to evolve into macrocracks that lead to the open circuit of printed stretchable conductors^14^. Meanwhile, the conductive networks for electron transfer are formed through the percolation of Ag flakes. However, the insulating surfactant introduced during the manufacturing of Ag flakes deteriorates the conductivity during both relaxed and stretched states. These two factors affect the realization of printed stretchable conductors with high conductivity, high stretchability, and low electrical hysteresis after cycling stretching. Especially, a low electrical hysteresis after cycling stretching is hard to achieve for printed composite conductors because of the viscoelastic property of elastomeric binders, cracking of the electrode, frictions among neighboring Ag flakes, and frictions between Ag flakes and binders^15,16^.

We designed a method for the low-temperature treatment of printed stretchable VegPU/Ag flake conductors to realize both high conductivity and electromechanical performance. After formulation of the ink and printing, the freshly printed uncured electrode is soaked inside the green sintering solutions containing organic acids and Cl^-^ to enable a room-temperature binder curing and sintering of the Ag flakes simultaneously, as shown in Fig. 1c. The curing initiated by the NIPS generated the porous structure while the sintering was performed by the partial removal of the insulating surfactant. NIPS relies on the high miscibility between non-solvent water and N, N-dimethylformamide (DMF) solvent, and thus the water can extract the DMF solvent and the polymer can be cured. The extraction of DMF leads to the polymer-rich matrix and polymer-poor pores. The porous binder can prevent the propagation of cracking during mechanical deformation (Fig. 1d–f and Supplementary Fig. 1). The organic acids and Cl^-^ work in the sintering solution synergistically to partially remove the insulating surfactant on the surface of Ag flakes, thus weakening the barrier for electron transfer and reducing the resistance variation during stretching. Figure 1g shows the performance comparison among VegPU/Ag with reported printable sustainable or biodegradable conductors, including solid flexible^17–21^, solid stretchable^22,23^, hydrogel electrical^24–28^, hydrogel ionic^29–33^, and plasticized conductors^34,35^. The VegPU/Ag conductor delivers the highest conductivity while maintaining superior stretchability. The combination of the sustainable elastomeric binder and designed treatment method endows the sustainable stretchable conductor with both high electrical conductivity and durability to mechanical deformation, especially for cycling stretching.

### Synthesis and characterization of sustainable VegPU

**Fig. 2:**
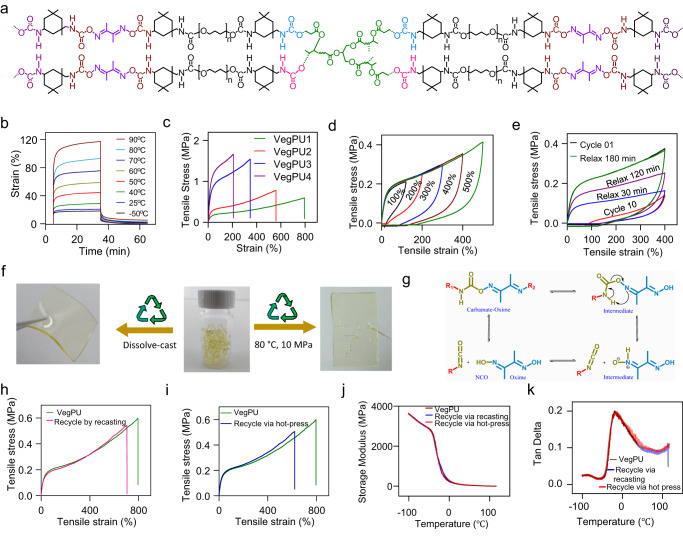
Sustainable VegPU binder synthesis and characterization. **a** Design of sustainable VegPU binder with superior mechanical and recycling properties. **b** Typical creep and recovery strain-time curves of VegPU under different applied temperatures. **c** Tensile stress-strain curves of VegPU1-4 with different molar ratios of SPO. **d** Cyclic loading-unloading curves of sustainable VegPU at 100 to 500% strains. **e** Cyclic loading-unloading curves and subsequent recovery cycles after a delay of 30 to 180 mins to investigate the self-recoverability ability of sustainable VegPU. **f** Recycled VegPU film through the solution casting and hot pressing. **g** The cyclic reaction mechanism of carbamate-oxime formation to a five-membered cyclic intramolecular charge transfer. **h** The stress–extension curve of recycled VegPU film via solution recasting. **i** The stress-extension curve of recycled VegPU film via hot-pressing. **j** The storage modulus curves of recycled sustainable VegPU films. **k** The loss factor curves of recycled sustainable VegPU films.

Figure 2a and Supplementary Figs. 2–6 depict the structure and facile synthesis of VegPU via step-growth polymerization with isophorone diisocyanate (IPDI), SPOs derived from vegetable oils, poly(tetramethylene ether) glycol (PTMEG), and dimethylglyoxime (DMG). The obtained VegPU binder, which has a renewable carbon content of >75–81%, could be used as the binder in composites for printable electronics. SPO is used as a SS due to its high crosslinking density, which enhances the thermal and mechanical properties of polyurethane films^36^. PTMG was used because of its flexible chain that can facilitate chain motion^37^. IPDI is chosen as the hard segment because urethane cyclohexyl ring steric hindrance facilitates covalent network restoration^38^. DMG is a chain extender that introduces reversible oxime bonds for dynamic covalent networks^39^. Furthermore, methyl groups in DMG prevent rigid segment crystallization and promote chain motion^40^. Consequently, VegPU contains three types of dynamic bonds: reversible carbamate-oxime intermolecular bonds, carbamate–carbamate bonds, and intramolecular hydrogen bonds. During mechanical deformation, the dissociation of the weaker hydrogen bonds can significantly dissipate energy, resulting in significant stretchability. The incorporation of a carbamate-oxime unit into VegPU confers recycling capability and facilitates the formation of multicomponent networks. The relatively sturdy carbamate-carbamate bonds guarantee structural inclusion for consistent mechanical characteristics. Overall, the synergistic effect of sustainable VegPU triple networks ensures both excellent mechanical and superior recycling properties.

The synthesis of SPO involves COBRA and epoxidized soybean oil (ESBO). The ^1^H nuclear magnetic resonance (NMR) and Fourier-transform infrared spectroscopy (FTIR) spectra confirm the successful preparation of COBRA as depicted in Supplementary Figs. 7-9. The ^1^H NMR peaks at 5.1–5.4 ppm and 4.2–4.3 ppm disappeared after the reduction reaction. Concurrently, the intensity of the new peak at 3.4–3.7 ppm increased, indicating that the triglyceride was completely reduced, which led to the formation of main hydroxyl groups (-OH). The degrees of epoxidation of soybean oil (SBO) were quantified using NMR and FTIR spectra in Supplementary Figs. 10 and 11. NMR spectrum illustrates that unsaturated fatty acids were transformed into epoxy groups labeled as diepoxides and monoepoxides, with signals having appeared at 3.06–3.16 ppm and 2.5–3.1 ppm (-CHOCH-CH_2_-CHOCH-)^41–43^. The FTIR of ESBO was followed by the C=C disappearance peak at 3011 cm^−1^ and the appearance of the new broad epoxy peak stretching vibration between 862–809 cm^−1^. Supplementary Figs. 12 and 13 depict the ^1^H-NMR and FTIR spectra of SPO for distinct intervals of carboxyl to epoxy group reactions. The peaks at 2.7–3.1 ppm representing the epoxy groups, decreased as reaction times increased. On the other hand, new peaks corresponding to 3° H atoms next to the newly formed ester groups were detected between 4.5–5.1 ppm (Supplementary Fig. 12). Supplementary Fig. 13 shows the epoxy groups in ESBO decreased, while a broad peak at 3395 cm^−1^ appeared, indicating that the epoxy groups in ESBO were ring-opened by the initiation of COBRA and sustainable hydroxyl (-OH) polyols were successfully formed^8,44^.

^1^H NMR, ^13^C NMR, and FTIR analysis confirmed the successful preparation of VegPUs by detecting IPDI, PTMG, SPO, and DMG in the polymeric backbones (Supplementary Figs. 14–16). As illustrated in Supplementary Fig. 15, the 7.2–7.5 ppm range had a proton peak that belonged to -CO-NH- VegPU urethane. Other proton peaks ranging from 1.0 – 4.5 ppm were associated with the -CH_2_ - and -CH_3_- in the VegPU’s main chain. Supplementary Fig. 17a, b depicts the FTIR spectra of a sustainable VegPU at various time intervals and carbamate-oxime ratios. As shown in Supplementary Fig. 17a, the peak at 2268 cm^−1^ was designated to the NCO groups of VegPU at fixed temperature and variable periods. The strength of the NCO peak at 2268 cm^−1^ reduces over time progressively with specific temperature, confirming the reaction between the -NCO group of IPDI and the -OH groups of PTMG, SPO, and DMG. After 360 min, there was almost no NCO prepolymer band at 2268 cm^−1^, indicating that the NCO and the -OH bonds had fully reacted. The following characteristic bands were found in the VegPU result: 3340–3356 cm^−1^ (N-H amide stretching), 3001–2800 cm^−1^ (anti-symmetric and symmetric aliphatic stretching), and 1725 cm^−1^ (C = O carbonyl stretching). The peak representing the N-O stretching vibration of oxime appears at 975–988 cm^−1^ ^45^ (Supplementary Fig. 17b). Dynamic mechanical analysis (DMA) in tensile mode was used to characterize the thermal properties of VegPUs (Supplementary Fig. 18). The glass transition temperature (*T*_g_) noted from tangents of the phase angles (tan delta) of VegPU1, VegPU2, and VegPU3 with 1.0, 0.8 and 0.6 molar ratios of SPOs were −20.42, 19.05, and −15.61, respectively, implying adequate crosslinking density for covalent carbamate bond transformation. After *T*_g_, storage modulus (*E*’) attains a rubbery plateau and then keeps dropping to nearly zero at high temperatures. Thermogravimetric analysis (TGA) in Supplementary Fig. 19 shows the weight loss of VegPU films and the derivative curves in nitrogen. First, labile carbamate-oxime groups decomposed from 240 to 350 °C. Chain scission in SPOs caused the second degradation from 350 to 500 °C.

Figure 2b and Supplementary Fig. 20 show creep and recovery strain-time curves of VegPU under different temperature and various applied stress, respectively. The elastic response of the VegPU films causes an instantaneous increment in strain in all creep curves. At a constant stress of 0.10 MPa, when the creep temperature ranged from −50 to 90 °C, strain increased from 19 to 117%. The creep-recovery behaviors and an almost immediate elastic recovery with a dramatic reduction in strain, once the stress was removed, occurred at all temperature stages, which indicated good elastomeric performance. When the stress was mounted from 0.10 to 0.22 MPa at 25 °C, the strain increased from 28 to 220% (Supplementary Fig. 20). The strain decreases rapidly to the initial elastic response when the stress is removed. This is due to the linear polyurethane linkages of the linked SPO in the soft segment of VegPU and also the oxime network in the hard segment^46^.

The tensile stress-strain curves of VegPU vary significantly with the amount of SPO crosslinks incorporated in the polymeric networks. As depicted in Fig. 2c, the tensile curves of VegPUs indicate that a higher SPO content of VegPU1 improved stretchability (797%) with an ultimate tensile strength of 0.66 MPa. However, the VegPU4 with a 0.4 molar ratio of SPO exhibited lower stretchability (205%) and a higher ultimate tensile strength (1.67 MPa) due to the higher cross-linking density of its starting SPO polyols. The cyclic loading-unloading tests showed high hysteresis (Fig. 2d). However, the VegPU film almost fully recovered its initial stress-strain behavior after an immediate unloading period, as evidenced by a crossover of the loading-unloading curves. These results pointed to the possible occurrence of mechanically dissociated DMG crosslinks to dissipate strain energy. Figure 2e shows continuous loading-unloading repeated 10 cyclic tests without waiting time at constant strains of 400% to further demonstrate the elastic performance of VegPU. The stretched VegPU film was then permitted to relax for 30 min (11th cycle), 120 min (12th cycle), and 180 min (13th cycle) at 25 °C until being strained then. Cycles 11 to 13 demonstrate excellent recovery of cyclic curves, which is close to the cycle, indicating the VegPU film’s mechanical recoverability. In addition, Supplementary Fig. 21 depicts the cyclic tensile tests of VegPU at a strain of 500%; after a 240 min resting period, the stress–strain curve fully recovered. We speculated that the identified excellent recovery in energy dissipative characteristics is due to the residual carbamate-oxime bonds, carbamate–carbamate unit orientation, dissociation of intramolecular H-bonds, and stress-softening behavior^46^. A notched test was done on VegPU (ASTM D412) for tear resistance evaluation (Supplementary Fig. 22). Despite a 2.5-mm notch of the cumulative width (5 mm), it would still withstand 345% tensile strain because of its dynamic oxime crosslinks premised on hydrogen bonds in the urethane structure. The comparison of synthesized VegPU with highly stretchable petrol-based PU and vegetable oil-based PU elastomers is shown in Supplementary Table 1. Though VegPU shows weaker stretchability compared with highly stretchable petrol-based PU elastomers, 797% elongation at break of VegPUs outperforms that of the most reported vegetable oil-based elastomers.

An interesting feature is the recyclability of the VegPU. Figure 2f shows that VegPU can be recycled via solution casting and hot-pressing methods. The recasting for recycling was performed by dissolving granules of the VegPU in the solvent and stirring for 5 h at 60 °C to produce a homogenous solution. After pouring the solution into the mold, it dried at 80 °C for 12 h. Hot-press recycling of VegPU film was hot-pressed for 10 min at 80 °C with 10 MPa pressure (Supplementary Figs. 23 and 24). Both methods ensured the complete reformation of oxime-carbamate bonds and no chemical degradation after recasting and remolding (Fig. 2g). The stress–extension curves of recycled VegPU from solution casting and hot-pressing are shown in Fig. 2h, i. Both tensile curves show that it is possible to achieve a similar mechanical performance after recycling VegPU films because of the reformation of oxime-carbamate bonds. Furthermore, as shown in Fig. 2j, k and Supplementary Fig. 25, we evaluated these same recycled VegPU films in terms of thermal responses using DSC and DMA analysis. Due to the plateau modulus directly proportional to polymer crosslinking, the E′ values of all three curves are the same, showing that VegPU networks can return to the same crosslink density as the first recycle after repeated recycling (Fig. 2j). The DMA tan delta and DSC *T*_g_ peaks of the three samples also correlated well, which shows that the glass transition behavior did not change in the recasting or molding process (Fig. 2k and Supplementary Fig. 25).

### Formation and conductivity of sustainable VegPU/Ag conductor

**Fig. 3:**
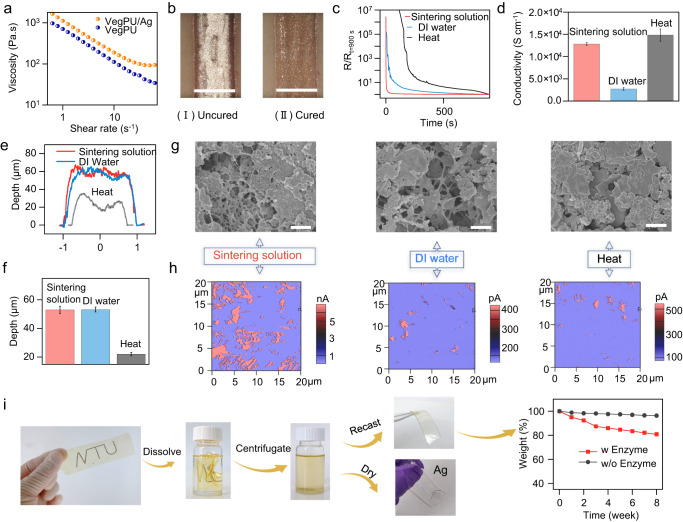
The electrical property and recycling of the sustainable VegPU/Ag conductor. **a** Viscosity curve of the VegPU/Ag ink. **b** Photo images of the printed VegPU/Ag electrode before and after sintering solution treatment, scale bar: 600 µm. **c** The resistance change of freshly printed VegPU/Ag electrode during sintering solution, DI water, and heat treatment. **d** Conductivity, surface profiles (**e**), thickness (**f**), SEM images (**g**), C-AFM (**h**) of sintering solution-, DI water-, and heat-treated VegPU/Ag electrode (*n* = 3). **i** The recycling of the VegPU/Ag conductor printed on VegPU substrate, including dissolving in DMF solvent, collecting of the VegPU and Ag flakes, and the degradation of VegPU at the end of life. Scale bar in (**g**): 2 µm.

The synthesized VegPU polymer resin was mixed with Ag flakes (SEM and particle size distribution are shown in Supplementary Fig. 26 to form an ink that delivered proper viscosity for screen printing. The fatty acid surfactant (with COOH group) on the surface of Ag flakes enables good dispersion in VegPU resin and the zeta potential is −47 mV, as shown in Supplementary Fig. 27. Figure 3a shows the viscosity curves of the formulated VegPU/Ag ink and pure VegPU resin, both presenting the typical shear-thinning property. The mixing with the Ag flakes increases the viscosity of the VegPU resin in the range that is suitable for screen printing^47^. The frequency dependence of the storage modulus (*G*’) and lost modulus (G”) of VegPU/Ag is shown in Supplementary Fig. 28. *G*’ is greater than *G*”, suggesting that the VegPU/Ag ink is liable to stay on the substrate after the deposition. After the printing, the ink is well attached to the surface of the substrate, as shown in Fig. 3b(I). After immediately immersing the sample in the sintering solution containing 100 mM NaCl and 17 mM lactic acid for 30 min, the geometric shape of the printed trace was not significantly affected, as shown in Fig. 3b(II). The 30 min sintering solution treatment at ambient temperature (20 °C) enables the printed trace to achieve desirable conductivity and attain a curing effect at room temperature by NIPS. The precise control of temperature, composition of sintering solution and treatment duration guaranteed the reproducibility of electrical performance.

NIPS can happen in the polymer solution after immersing in a coagulation bath containing a poor solvent. The solvent and non-solvent must be miscible, so the polymer can be solidified through the solvent-nonsolvent exchange. This method has been widely used in preparing membranes for separation processes in biotechnology, chemical industries, and environmental science^48^, but has rarely been applied to printed electronics. The high miscibility between non-solvent water and DMF solvent allows the extraction of solvent from water and the cure of the polymer solution. Inductively coupled plasma mass spectrometry (ICP-MS) was utilized to analyze the possible release of Ag into the sintering solution, as shown in Supplementary Fig. 29. The Ag released into the sintering solution during the treatment can be in the form of either Ag^+^ or Ag flakes. After 30 min of immersion, the total released Ag accounts for 0.33 wt% of the printed electrode, with 0.04 wt% in the form of Ag^+^ and 0.29 wt% in the form of detached Ag flakes, indicating the loss of Ag from the VegPU/Ag conductor is minimal.

Initially, with the solvent, the freshly printed traces are not conductive, as the volume proportion of Ag flakes is quite low (~10.5 vol%) and it is hard to form the electron transfer networks. As the solvent was removed by either NIPS or heat treatment, Ag flakes would be packed at a higher density that exceeded the electrically conductive threshold, and electron conductive pathways can be formed by either the tunneling effect or direct contact among Ag flakes. The resistance changes of the electrodes treated by sintering solution and deionized (DI) water were shown in Fig. 3c. Meanwhile, the electrode produced by heat treatment (80 °C) was also studied as a comparison. The NIPS in both aqueous solutions (sintering solution and DI water) displayed a more rapid formation (30 s) of conductive pathways than that of heat treatment (180 s), suggesting that NIPS is time-saving and effective in curing printed ink.

The final conductivity of the electrodes cured by different methods is shown in Fig. 3d. The heat-treated electrode owns the highest overall conductivity of 14,840 S cm^−1^ among the three types of electrodes. This can be explained by the highest volume density of Ag flakes inside the electrode cured by heat, as explained in the following. The surface profile of the three electrodes is shown in Fig. 3e, f. With the same parameters for the printing stencil and the same ink, the heat-treated electrode has the lowest volume with a height of around 22.04 µm and a width of 1.483 mm, whereas the volume of the resultant composite prepared with the sintering solution (52.96 µm and 1.998 mm) and DI water (53.19 µm and 1.969 mm) is much higher. The total volumes of porous sintering solution and DI water-treated electrodes are 2.24 and 2.20 times higher than that of the heat-treated electrode, suggesting that the NIPS-induced pores in the binder^48^ significantly increase the total volume of the electrode and lower the volume density of the Ag flakes. Thus, the average distance among Ag flakes in porous electrodes was more than 3 times of the heat-treated dense electrode, leading to higher resistance in porous electrodes when compared with the heat-treated electrodes.

The much bigger geometrical size of the sintering solution- and DI water-treated electrodes can be ascribed to the water-induced phase separation that produces the pores in the VegPU binder. The extraction of solvent leads to the polymer-rich matrix and polymer-poor pores, as shown in the top-surface and cross-section scanning electron microscopy (SEM) images of the VegPU films cured by different methods in Supplementary Figs. 30 and 31, respectively. The polymer gradient in the vertical direction caused by the coexistence of nonsolvent absorption, solvent-nonsolvent exchange, and solvent evaporation, leads to an asymmetric structure with a top thin dense layer on the microporous sublayer in the NIPS-induced VegPU. In terms of the Ag flakes and VegPU composite, the sintering solution and DI water curing produced pores in the binder of the printed electrode, as shown in Fig. 3g, whereas no porous binder appeared in the heat-treated electrode. These results suggest that the presence of water is the key factor in the formation of the porous binder inside the sustainable VegPU/Ag electrodes. After curing, Ag flakes are well dispersed in the VegPU binder, as shown in the EDX of the sintering solution-treated electrode in Supplementary Fig. 32. Meanwhile, the surface of the Ag flakes of the sintering solution-treated electrode is rougher than that of the DI water-treated and heat-treated electrodes, as shown in Supplementary Fig. 33. This can be ascribed to the sintering reaction between the sintering solution (which contains Cl^-^ ions) and Ag flakes^49^. The SEM images of the Ag flakes (Supplementary Fig. 34) after being treated with sintering solution, DI water, and heat further prove this point.

Notably, the electrode treated with sintering solution has much higher conductivity (12,833 S cm^−1^) than that of DI water-treated electrodes of 2714 S cm^−1^. This is probably due to the sintering reaction on Ag flakes induced by Cl^−^ and H^+^ in the sintering solution. The Cl^−^ and H^+^ can work synergistically to partially remove the surfactant on the Ag flakes and facilitate the redeposition of Ag nanoparticles on the surface, thus enhancing the conductivity of the Ag flakes electrode. For the DI water-treated electrode, the lubricant surfactant still covers the surface of Ag flakes, impeding the electron transfer among Ag flakes. This can be validated by a higher remaining weight and a lower C/Ag ratio of Ag flakes treated by sintering solution in thermogravimetric analysis (TGA) and X-ray photoelectron spectroscopy (XPS), respectively^49^. The conductive atomic force microscopies (C-AFM) of three electrodes are shown in Fig. 3h and Supplementary Fig. 35. The surface of the sintering solution-treated electrode is more conductive than that of the DI water-treated and heat-treated electrodes. To further demonstrate the effect of the sintering reaction, we retreat the DI water- and heat-treated electrodes with the sintering solution. As shown in Supplementary Fig. 36, the sintering solution retreatment endows a higher conductivity than pristine electrodes. For DI water-treated electrode, the conductivity was increased from 2714 to 11,306 S cm^−1^, while the conductivity of the heat-treated electrode was even enhanced to 21,324 S cm^−1^, suggesting the effectiveness of the sintering solution in improving the conductivity of the electrodes.

The sustainable VegPU/Ag electrode can be recycled by dissolving it in the solvent. Then the Ag conductive fillers can be collected by centrifugation and the VegPU binder can be reused by casting a new thin film, as shown in Fig. 3i. When the recycled VegPU was applied in the formulation of the ink, as shown in Supplementary Fig. 37a, b, the conductivity and stretchability of the VegPU/Ag electrode deliver a minor deviation. However, the recycled Ag flakes aggregated with each other due to the partial removal of surfactant and enhanced contact among Ag flakes during the sintering solution treatment, as shown in Supplementary Fig. 37c. The reproduction of Ag conductive fillers from collected Ag flakes is required for recycling purposes. In addition, the biodegradation of VegPU was also tested in the presence of a lipid enzyme. The enzymatic degradation was progressing with the reaction time, after 8 weeks, the weight loss is around 19.2%. By comparison, pure water cannot significantly degrade the VegPU. The photo images of the VegPU before and after the reaction with lipase and pure water solutions are shown in Supplementary Fig. 38. Compared with a pure water solution, the enzyme solution has changed the surface of the VegPU, further suggesting that the enzyme could decompose the VegPU. The lipase could penetrate the VegPU through the sustainable soft domain and catalyze the decomposition of ester groups^50,51^.

### Electromechanical performance of sustainable VegPU/Ag conductor

**Fig. 4:**
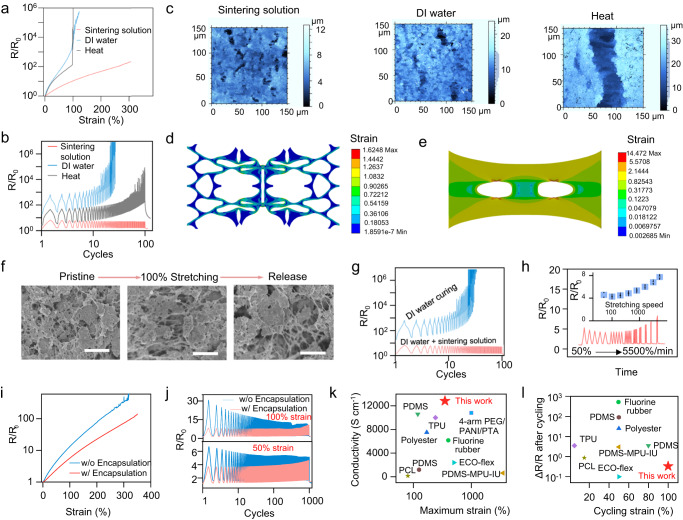
The electromechanical performance of VegPU/Ag conductors. The resistance changes upon elongation (**a**) and during 50% cyclic stretching (**b**). **c** The confocal images of electrodes with the presence of 100% strain; **d**, **e** the strain distribution in porous VegPU and dense VegPU with precuts upon 100% strain, respectively. **f** The SEM images of sintering solution-treated VegPU/Ag before, during, and after 100% stretching, scale bar: 3 µm. **g** The resistance changes of DI water-treated VegPU/Ag electrodes with subsequent sintering solution treatment during 50% cycling stretching. **h** The resistance changes of sintering solution-treated electrode at 50% cycling stretching with varied stretching speed (*n* = 5). **i**, **j** The resistance charges of VegPU-encapsulated VegPU/Ag electrode during single stretching and 1000 cycles stretching (50% and 100%), respectively. w/o and w/ represent without and with, respectively. The performance comparison of sustainable porous VegPU/Ag conductor with reported petrol oil-based elastomers/Ag conductors, including maximum strain–conductivity (**k**) at the relaxed state and cycling strain–electrical hysteresis (**l**). The reported conductors utilized fluorine rubber^59^, polyester^61^, PDMS^62,65^, TPU^60^, PDMS-4,4′-methylene bis(phenyl urea)-isophorone bis urea units (PDMS-MPU-IU)^63^, Ecoflex^64^, 4-arm polyethylene glycol/poly-aniline/phosphotungstic acid (4-arm PEG/PANI/PTA)^66^, polycaprolactone (PCL)^12^ as binders.

Upon stretching, the sintering solution-treated electrode delivers a much higher stretching limit and a lower resistance increment than that of the heat-treated electrode and DI water-treated electrode, as shown in Fig. 4a. The sintering solution-treated electrode can be stretched around 300%, which is much higher than that of the heat-treated electrode (98.3%) and DI water-treated electrode (122.4%). Also, the resistance change R/R_0_ of the sintering solution-treated electrode is much lower compared with the other two electrodes. The cycling stretching is one of the biggest challenges for the printed stretchable Ag flake conductors, especially for electrodes with a high loading of Ag flakes conductive fillers and/or the utilization of physically crosslinked polymers as the binders^4,52^. Most of the reported printed electrodes based on physically crosslinked polymer underwent significant resistance increases during the cycling stretching, though they have outstanding first-time stretching performance^33,34^. We evaluated the resistance change of three electrodes under the 50% cycling stretching, as shown in Fig. 4b. With a ~59.9 vol% of Ag flakes in VegPU/Ag composite, the cycling stretching has detrimental effects on the heat-treated and DI water-treated electrodes, whereas the sintering solution-treated electrode delivered a significantly reduced resistance change. The electrical hysteresis (Δ*R*/*R*_0_) after 100 cycles is around 0.281 (with 300 s rest after stretching for all cases), which is much lower than that of the DI water-treated and heat-treated electrodes. The further increase (liable to cracking) or decrease of the Ag flakes (less percolation networks) proportion led to the deteriorated cyclic stretching performance, as shown in Supplementary Fig. 39. Therefore, the weight ratio of Ag flakes and VegPU resin 5:4 was selected in all experiments.

Figure 4c shows the confocal images of the sintering solution-, DI water-, and heat-treated electrodes with the presence of 100% of strain, respectively. Only microcracks appeared on the surface of the sintering solution- and DI water-treated electrodes, with lengths less than 25 and 35 µm, respectively, whereas macrocracks (2 mm, across the width of the printed pattern) were generated on the heat-treated electrode, suggesting that the porous binder helps prevent the evolution of cracks upon deformation. The finite element analysis was applied to illustrate the role of pores in weakening/preventing the propagation of cracks. We simplified the model by analyzing the cracking properties of porous VegPU binder, as the binder holds the conductive fillers together and maintains the complete shape of the conductor. A generalized Maxwell viscoelastic model was used to qualitatively describe the mechanical properties of the VegPU^53^ and the shear stress relaxation of VegPU at ambient temperature (20 °C) was utilized as the input data (Supplementary Fig. 40). The volume proportion of the rigid Ag flakes in the formulated ink is quite high and thus there are many defects in the Veg-PU binder matrix. Here, we utilize two pre-cuts (cracks) to simulate the presence of defects in the practical situation. When considering the presence of pre-cuts of the same size (Supplementary Fig. 41), compared with the dense elastomer film, the voids in the binder are very helpful in blunting the crack tips and changing or deflecting the direction of the crack path more along the strain loading direction. As shown in the strain distribution of porous and dense VegPU with the presence of 100% of strain in Fig. 4d, e, the maximum strains of the porous and dense VegPU are 162.5% and 1447.2%, respectively. The crack tips in the dense film are prone to grow and finally led to the break of printed electrodes. The voids serve as the sink for the dislocations of cracks and thus delay the growth of the cracking during stretching and increase the resistance of the binder to fracture failure^54,55^. The SEM images of the sintering solution-, DI water-, and heat-treated electrodes before and during 100% stretching also prove this phenomenon, as shown in Fig. 4f and Supplementary Figs. 42 and 43, respectively. The whole loading/unloading process involved the reorganization of Ag flakes.

However, even without macrocracks during stretching, the DI water-treated electrode still delivers much more deteriorated electrical performance upon single and cycling stretching than that of the sintering solution-treated electrode, suggesting that the sintering reaction and porous binder contribute synergistically to the significantly enhanced durability of stretching deformation. The conductive network constructed by percolated Ag flakes is responsible for the electrical performance. The sintering reaction can remove the insulating lubricant and even connect some adjunct Ag flakes, thus reducing the tunneling potential barrier of the Ag flakes. Even though the DI water-treated electrode has a similar less-cracking surface to that of the sintering solution-treated electrode, the resistance increase upon stretching is much more prominent at 100% strain and 50% cycling strain.

The deformation of conductive fillers is neglected during stretching, as the modulus of Ag conductive fillers is much higher than that of VegPU. So, the resistance change during stretching is mainly caused by the interparticle separation. The resistance change under the strain can be described by the following Eq. (1)^56^:

1 $$\frac{R}{{R}_{0}}=\frac{s}{{s}_{0}}\exp \left[\frac{4\pi \sqrt{2m\varphi }}{h}(s-{s}_{0})\right]$$

In which, *R* and *R*_0_ are the resistance before and after the tensile deformation, *s* and ${s}_{0}$ are the average interparticle distance of the stretched and original states, respectively, *m* is the mass of an electron, $\varphi$ is the height of the potential barrier among conductive fillers, and $h$ is the Plank’s constant. The sintering solution and DI water-treated electrodes have the same ink formulation and similar geometrical parameters (average interparticle distance). The difference is that Ag flakes in the sintering solution-treated electrode with less surfactant, as the sintering solution can partially remove the surfactant, leading to a reduced tunneling potential barrier height that can significantly mitigate the resistance change when the electrode was stretched. Meanwhile, the percolation produced by the sintering reaction may also contribute to the electron transfer upon stretching, because of the strong adhesion among contacting Ag flakes^57^. This effect was fully proved by retreating the DI water-treated electrode with the sintering solution and then checking the resistance change upon stretching. Compare with the original DI water-treated electrode, beyond with enhanced conductivity (4.17 times), further retreatment by the sintering solution can dramatically increase the electrode’s maximum stretchability (from 103% to 246%) and durability to cycling stretching (Fig. 4g and Supplementary Fig. 44).

The combination of porous structure in binder and sintering reaction induced by the curing of sustainable sintering solution enables the printed electrode with high durability to tensile mechanical deformation. The porous structure can effectively prevent the evolution of the cracks, while the sintering solution can decrease the length of the tunneling potential barrier and realize a relatively low resistance change upon stretching. The function of the porous binder in enhancing the stretchability was also supported by retreating the heat-treated electrode with a sintering solution. As shown in Supplementary Fig. 45, the stretchability of heat-treated electrode without porous binder was even reduced from 98.3% to 42.9%, though the conductivity was increased by sintering reaction (1.44 times), suggesting the great capability of porous binder in accommodating the applied strain. Except for the lactic acid, other sustainable organic acids (such as citric acid and acetic acid) are also feasible in enhancing the stretchability of the VegPU/Ag electrode when coupled with NaCl. As shown in Supplementary Fig. 46, the sintering solutions containing NaCl and either lactic acid, citric acid, or acetic acid have a similar capability in reducing the resistance change upon stretching.

The stretching rate-dependence behavior is also commonly observed for printed Ag-based composite electrodes, as the high speed for stretching may increase the hysteresis of the elastomeric binder and accelerate the cracking propagation^58^. The strain rate-dependent property of elastomeric binder makes the resistance increment sensitive to how fast the strain is implemented, which is unfavorable as the stretching speed is variable in some practical applications. Due to the good bonding between Ag flakes and VegPU, as shown in Supplementary Fig. 47 (the SEM images of VegPU after 1000 cycles of 50% stretching), the cyclic stretching speed changes the silver network and reorganization of silver networks during stretching and the release of the strain, respectively, through the dragging of viscoelastic VegPU. The resistance change of the printed electrode with 50% strain at different strain rates was shown in Fig. 4h. When the strain rate was increased more than 100 times, from 50% to 5500% min^−1^ (from 1 to 110 cm min^−1^), the maximum *R*/*R*_0_ only increased from 4.6 to 7.7 (inset of Fig. 4h) and the resistance can return to around the initial resistance. The sintering solution-treated electrode with a porous structure has a small train rate dependence and may significantly widen its application in printed stretchable electronics.

The printed electrode may require encapsulation in some applications to protect itself and avoid short-circuiting. Here, we choose the same sustainable VegPU as the encapsulation layer. As shown in Fig. 4i, the encapsulated electrode delivers much lower resistance changes than the original electrode and the stretchability can even reach around 350%. This can be ascribed to the effect of the top sustainable VegPU layer in protecting the conducting network upon stretching. During the 50% and 100% cycling stretching, as shown in Fig. 4j, the encapsulated electrode also shows better performance. The maximum relative resistance change *R*/*R*_0_ is around 10.7 during 1000 cycles of 100% stretching, suggesting its great durability to repeated mechanical deformation. The SEM image of the encapsulated electrode was shown in Supplementary Fig. 48. Even though the porous sustainable VegPU on the top part of the printed conductors may be dissolved by the solvent in the encapsulating resin, the inner network remains the porous structure.

Stretchable textile electronics are attracting intensive attention in wearable technology, as textiles are essential for human beings and always have intimate contact with human skin. However, the realization of a reliable printed stretchable conductor on textiles is challenging as the surface of the textile is uneven and deformation can be fully transferred to the printed traces by the movement of the fibers. Our sustainable polymer-based electrode is also applicable to stretchable textile substrates. As shown in Supplementary Fig. 49, the sintering solution-treated electrode on the textile has an *R*/*R*_0_ of 4.14 at 120% strain, while the heat-treated electrode shows an *R*/*R*_0_ of 7.62. The cyclic performance was even better than that of the heat-treated textile electrode, showing a maximum *R*/*R*_0_ of 3.23 during 50% cycling stretching.

The changes in resistance of one freshly printed VegPU/Ag electrode during the sintering solution treatment and thereafter 50% cycling stretching can be found in supplementary movie 1. The VegPU/Ag electrode can be rapidly cured by the sintering solution and delivered durability to the cyclic stretching. After three weeks, the resistance and stretchability are stable without significant change, as shown in Supplementary Fig. 50. The performance comparison of the printed electrode with the reported literature based on Ag flakes is shown in Fig. 4k, l, and Supplementary Table 2^12,59–66^. The conductivity of sustainable VegPU/Ag is prominent compared with reported stretchable Ag flakes conductors using traditional petrol oil-based elastomeric binders, while the maximum stretchability is 350% which is higher than conductors using thermal plastics polyurethane (TPU) and polydimethylsiloxane (PDMS) (Fig. 4k). In terms of cyclic stretching, which is the common challenge for most printed stretchable conductors, the VegPU/Ag shows distinctive performance over reported stretchable conductors (Fig. 4l). Our sustainable and stretchable conductor combing with well synthesized VegPU elastomeric binder and a designed curing method shows superior overall performances including conductivity, highest stretchability, and cycling stretchability, paving the way for its wide applications.

### Application in soft smart gripper for fruit maturity sensing

**Fig. 5:**
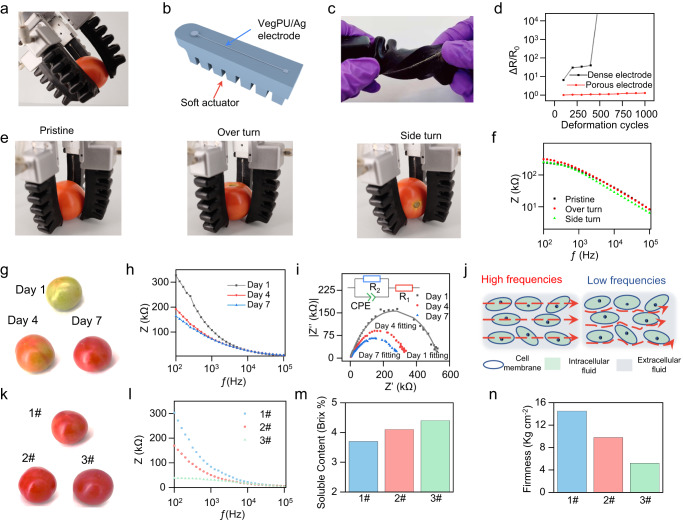
Soft smart gripper for fruit maturity sensing. **a** The pneumatic soft grippers compose four actuators for the gripping of a tomato. **b** The schematics of the VegPU/Ag electrode on a soft pneumatic actuator. **c** The twisting deformation of a soft pneumatic actuator. **d** The resistance changes of VegPU/Ag electrode after cycling twisting deformation. **e**, **f** The different placements/gestures and measured impedance of the gripper when gripping a tomato, respectively. The photo images (**g**), Bode impedance curves (**h**), and Nyquist impedance (**i**) were acquired by the soft gripper of one tomato during 7 days of storage. **j** Schematics of ions movement inside the tomato tissue at high and low frequencies. The photo images (**k**), Bode impedance curves acquired by the soft gripper (**l**), soluble content (**m**), and firmness (**n**) of three tomatoes.

To demonstrate the application of our sustainable VegPU/Ag electrode, we designed a stretchable electrical impedance spectroscopy (EIS) sensor on pneumatic soft grippers for measuring the ripening level of the fruits, as shown in Fig. 5a. The EIS can indicate the physio-chemical status of fruits and vegetables at an early stage in a non-obstructive way, thus making it suitable to integrate with soft grippers for automatic fruit monitoring, picking, and sorting^67^. The validation of using EIS to monitor the ripening level of fruit and vegetables, such as bananas^68^, tomatoes^69^, mango^70^, strawberries^71^, avocado^72^, and apples^73^ has been reported in previous works. However, these works utilized rigid electrodes for measurement, limiting the combination with soft robotics for automatic gripping and testing. We integrated four pneumatic actuators with stretchable sensors to form a gripper for simultaneous gripping and sensing, as shown in Fig. 5b. Due to the softness of grippers and repeating movement during the deployment, the soft actuators are always subjected to cyclic deformation. Herein, the capability of the sensor attached to the actuator to endure cyclic twisting was shown in Fig. 5c. Due to the much bigger dimensional size than that of thin film electrodes, the twisting of actuators causes the compression, stretching, and twisting deformation on the surface electrode. After 500 cycles of 180° twisting, the heat-treated electrode lost the conductivity, while the sintering solution-treated electrode with porous structure in a binder and sintered Ag flakes only experienced a 1.25 times increment, as shown in Fig. 5d, suggesting the robustness of the printed VegPU/Ag in enduring the cyclic deformation.

Herein, we utilized tomato as one example to show the capability of the soft sensor in detecting the ripening level of fruits. The soft gripper was used to grip the tomato with different placements on the table to test the effect of uncertain contact between the tomato and the gripper on testing results, as shown in Fig. 5e. The impedance curves tested on different contact points on the tomato surface show a minimal difference (Fig. 5f), owing to the almost symmetric shape of the tomato and the soft sensor that guarantees good contact with the curved tomato surfaces. The effect of contact points can be further eliminated by optimizing the gripper configuration and Young’s modulus^74,75^. In practical usage, the gripper may experience intensive movement during automatic picking and sorting. The impedance curve of the tomato during shaking is close to that of the static status, as shown in Supplementary Fig. 51, and Supplementary Movie 2 shows the stable impedance at 1 kHz during shaking, suggesting that our soft impedance sensor can undertake the task during dynamic movement of the tomato.

With one week of storage, the color of the raw tomato changed from green to red and the impedance drops accordingly from 327 to 164 kΩ at the frequency of 100 Hz, as shown in Fig. 5g, h, respectively. The EIS and fitting data of one tomato during the aging in 1 week were shown in Fig. 5i. The Cole model fits well for the construction of the equivalent circuit. The cellular membrane with dielectric interface behavior is represented by a capacitor (*C*) and the flowing currents in the intracellular and extracellular fluids are modeled by *R*_1_ and *R*_2_, respectively. The diagram illustration of the biological structure of the tomato tissue is illustrated in Fig. 5j. When a high-frequency current passes the tomato, the impedance consists of parallel of *R*_1_ and *R*_2_ is mainly dominated by the intracellular fluid (*R*_1_), as the cell membrane is short-circuited, and the current can transfer through the intracellular fluids which have a higher concentration of electrolytes than the extracellular fluid. However, the capacitive cell membrane blocks the transfer of current through the intracellular fluids when a low-frequency current is applied. Thus, the impedance is mainly determined by the extracellular fluids, of which the resistance is much higher than that of intercellular fluids. The change of *R*_1_ and *R*_2_ in Supplementary Fig. 52 can support the changes in the tomato during aging. The intracellular impedance experienced a gradual increase, while extracellular impedance decreased with the aging process, because of the deterioration of the cell membrane associated with senescence. The deteriorated cell membrane could not maintain the previous cell turgor and thus the homeostatic process leads to the absorption of water from extracellular fluids. The dilution of intercellular fluids causes the reduction of conductivity and water loss leading to the enhanced conductivity of extracellular fluids. Meanwhile, the loss of water during storage also resulted in an increased concentration of electrolytes in extracellular fluids. Our gripper can monitor the aging process of the tomato, suggesting its potential use in automatic fruit sorting and picking.

The capability of the gripper in detecting the physiological level of different tomatoes with similar colors was also studied. Though three tomatoes are of similar colors (Fig. 5k), the impedance of tomato 1 is much higher than that of the other two tomatoes, as shown in Fig. 5l. This difference did not come from the weight variation, as shown in Supplementary Fig. 53, 3 tomatoes with a similar weight, and tomato 1 is the lightest among the three tomatoes. To further build the correlation of EIS data with the inner physiological status, the softness and soluble content of three tomatoes were tested by an obstructive fruit hardness tester and a total soluble solids refractometer (Supplementary Fig. 54), respectively. Two parameters have been applied to indicate the ripeness of the fruit^76^. As shown in Fig. 5m, n, the hardness has the same trend as impedance, while the total soluble content has the opposite trend. The hardness is mainly related to the proportion of starch transferring to sugar during aging, cell wall ultra-structure change, and the metabolism-caused softening of pulp^77^. The testing of soluble contents (sugars, acids, and other components) is a fast, cheap, and widely used way to indicate the sugar level of fruits, even though the result is imprecise somehow^76^. Further systematic studies including the tight correlation between measured impedance and fruit quality, the miniaturization of analyzers, and data analysis are required for the wide application of soft EIS grippers in smart agriculture.

## Discussion

In summary, we realize the printed sustainable conductor with high conductivity and overall superior electromechanical properties through the combination of synthesized sustainable recyclable Veg PU elastomeric binders and an invented sintering solution curing method. Castor oil-derived bifunctional ricinoleic oil and epoxidized soybean oil were utilized to construct polyols for the dynamically crosslinked covalent networks in sustainable recyclable Veg PU. We endow the printed sustainable VegPU/Ag conductor with a high electromechanical performance by soaking the freshly printed conductor in the sintering solution containing a sustainable organic acid and Cl^-^ to realize the porous binder and sintering of Ag flakes. The porous VegPU binder that can weaken/impede the growth of cracks upon stretching and sintering reaction can enhance the electron transfer capability at both relaxed and stretched states. Two factors work synergistically to enhance the overall electromechanical performance of printed porous VegPU/Ag conductors. The performance of the VegPU/Ag conductors can be affected by the particle size, which requires an additional study to further increase the electromechanical performance. Meanwhile, future work on avoiding the use of toxic organic solvents will further enhance sustainability. A stretchable impedance sensor on the soft gripper that can monitor the maturity level of fruit non-obstructively was printed to demonstrate the application of our printed porous sustainable VegPU/Ag conductor. The scalable printing manufacturing and great overall performance would significantly expand the widespread of our sustainable stretchable conductors in the design of sustainable soft electronics.

## Methods

### Materials

Poly(tetramethylene ether) glycol (Mw = 1000 g/mol, 98%) and castor oil (CO, Mw = 933.45, 90% ricinoleic acid) have been obtained from Sigma-Aldrich and refluxed for 3 h at 120 °C before being used. Soybean oil (SBO, Sigma-Aldrich), aliphatic isophorone diisocyanate (IPDI, 98%, Alfa Aesar), formic acid (HCOOH, ≥95%, Sigma-Aldrich), and N, N-dimethylformamide (DMF, 99.5%, Thermo Scientific™) were used without additional distillation. The diethyl ether (Et_2_O, ≥99.5%), dibutyltin dilaurate (DBTDL, 95%), DMG (≥99%), silver flakes (<10 µm), lactic acid (~90%), sodium chloride (≥99%), acetic acid (≥97%), citric acid monohydrate (≥98%), lipase (from Candida rugosa) were purchased from Sigma-Aldrich and used in their original forms. Stretchable TPU substrate and textile Lycra Shiny Milliskin Nylon Spandex Fabric was purchased from Spandex World. Inc, USA, and Dongguan Jiaxin Plastic Materials, respectively. Cambridge Isotope Laboratories Inc. provided deuterated solvents for NMR characterization. Double-deionized water was used throughout the study.

### Synthesis of COBRA

Bifunctional ricinoleic acid could be acquired through either saponification or fractional distillation of castor oil that has been hydrogenated. Supplementary Fig. 3 illustrates the synthesis of COBRA. Initially, required quantities of castor oil were saponified into bifunctional ricinoleic acid by heating a sodium hydroxide (NaOH) mixture at 80–85 °C for 4 h. The COBRA solution was then neutralized using diluted hydrochloric acid (HCl). The organic phase has been filtered after neutralization by washing with double-distilled water (*dd*H_2_O). The dissolution–decantation process was routine four times, and the product was dried over magnesium sulfate (MgSO_4_). The majority of the bifunctional fatty acids in castor oil are ricinoleic acid (88–91%), and additional acids are present, including linolenic, linoleic, and oleic acids.

### Synthesis of ESBO

In a typical experiment, a measured quantity of soybean oil (0.5038 mol) was processed at 70 °C for 1 h in a 250 mL round-bottle flask with a thermometer, and an oil bath. Thereafter, a mixture of hydrogen peroxide (H_2_O_2_, 0.2855 mol) and formic acid (HCOOH, 0.0570 mol) was introduced to the soybean oil with the catalyst at a syringe pump rate of 0.20 cm^3^ min^−1^. The resultant mixture was stirred periodically and kept at the same temperature of 70 °C for 5 h. After the addition of the oxidant mixture, the reaction proceeded for the preferred duration of time. Throughout the reaction, samples were taken at regular intervals. The samples were quenched and centrifuged to separate the organic layer from the aqueous medium. The samples were washed with water until they were acid-free before being analyzed. Supplementary Fig. 5 illustrates the synthesis of ESBO.

### Synthesis of SPO based on ESBO and COBRA

Firstly, COBRA was placed in a flask with a mole ratio of the carboxyl group of 1.5, stirred with a mechanical stirrer, and kept at 150 °C in an N_2_-free environment. Then, ESBO at a mole ratio of 3 to the epoxy group was added drop by drop while vigorously stirring. After being mixed fully, the mixture was kept at 140 to 180 °C overnight with the constant stirring condition. To quench the reaction, 30% ammonia in water was added to the solution mixture. After the reaction was complete, the finished SPO products were obtained with Et_2_O and washed at least five times with double-distilled water. After drying the precipitate with MgSO_4_, it was filtered. After removing the organic solvent with Rotavapor and vacuum, the clear viscous pale yellow SPO was obtained. The yields of SPO were approximately 90%. The preparation of representative SPO is depicted in Supplementary Fig. 6.

### Synthesis of vegetable oil-based polyurethanes (VegPU)

A series of VegPU were synthesized from SPO and PTMG with IPDI at a molar ratio of 0.8:0.5:1.5 (-OH/-NCO). First, the PTMG-IPDI-based NCO-terminated precursor was prepared by the PTMEG and IPDI chemical reaction with a 2:3 mole ratio at 75 °C–80 °C under a nitrogen condition for 6 h. The PTMEG-IPDI precursor (4 mmol) and SPO (8.03 mmol) were solubilized in anhydrous DMF (20 ml) and were left to react for 4 h at 80 °C. The reacting solution was then given one drop of DBTDL and stirred for another 30 min. Subsequently, dimethylglyoxime (DMG) (4.04 mmol) as a chain extender was diluted in DMF (5 mL), and the resulting solution was added into the reaction system that was further maintained at 65 °C for 2 h. The resultant polymer solution was then placed into a glass mold (length × width: 100 mm × 50 mm) and dried in an 80 °C oven for 12 h. Lastly, specific dimensions of the VegPU films were cut for thermo-mechanical testing. Supplementary Fig. 2 depicts the synthesis of a representative VegPU from SPO. To examine the effect of SPO content on reactivity, 0.4, 0.6, 0.8, and 1.0 molar ratios of SPO were expanded to the urethane prepolymer reaction mixture. The resulting VegPU with 1.0, 0.8, 0.6, and 0.4 molar ratios of SPO is designated as VegPU1, VegPU2, VegPU3, and VegPU4 respectively.

### VegPU characterization

To understand the molecular structure of the sustainable VegPU, ^1^H and ^13^C NMR spectroscopy (400 MHz Bruker) and FTIR (PerkinElmer) were used. At room temperature, ^1^H NMR and ^13^C NMR were done using tetramethylsilane (TMS) as an additional source and deuterated solvents as an internal lock reference. TGA (TA Instruments Q50) and DSC (TA Instruments Q20) were used to figure out how the VegPU film behaved in terms of heat. Ten milligrams of VegPU were put in a platinum pan and burned at a thermal heating rate of 10 °C min^−1^ as the temperature went from room temperature to 700 °C. DMA analysis was done on a dynamic mechanical analyzer (DMA Q800), and specimens were tested in a liquid N_2_ environment ranging from 80 °C to 170 °C at a heating flow rate of 10 °C min^−1^. Both the stress relaxation and creep tests used the same DMA Q800. All mechanical stress-strain was performed at room temperature using a Materials Testing Systems (MTS, C43) criterion model 43 mechanical detector with a load cell bearing of 500 kN and a tensile strain rate of 100 mm min^−1^. A Physica MCR 501 rheometer was used to measure the dynamic viscoelastic rheological properties (Anton Paar). Most analyses were done at room temperature and 2 Hz with an 8 mm probe (PP08150, Anton Paar) with a 0.3 mm disparity.

### The formulation, printing, and curing of the VegPU/Ag ink

The VegPU resin was mixed with Ag flakes at a weight ratio of 4:5 by 3 h shaking on a vortex (MX-S, Dlab). The printing pattern was designed by Auto CAD software and printed by a stainless-steel stencil (Micro Tech Technology, Shenzhen, China) with a thickness of 100 µm produced by laser cutting. Then, the freshly printed samples were immediately immersed in the curing solution containing 100 mM NaCl and 17 mM lactic acid. After 30 min soaking, the printed electrodes were dried in the oven at 60 °C for 3 h. The water- and heat-treated electrodes were produced by putting the freshly printed electrodes in the DI water for 30 min and in the oven at 80 °C for 4 h, respectively.

### The characterization of the VegPU/Ag electrodes

The photo image of the freshly printed electrode before and after the sintering curing was performed on an upright microscope (Olympus BX51). The geometrical parameters including the thickness and width after printing were tested on the surface profile D500 (KLA). The conductivity of the printed electrodes was tested by the four-probe method on a multimeter (DAQ6510, Keithley). JEOL 7600 and C-AFM (Asylum Research Cypher S) were used to test the surface conductivity and SEM images, respectively. The stretching test of printed VegPU/Ag electrodes (2 mm × 2 cm) was performed on a motorized force test stand (ESM303, Mark-10) with a speed of 2 mm min^−1^ and the corresponding resistance change was tested by a multimeter. The cycling stretching speed is 60 mm min^−1^ and the stretching rate dependence was tested with speeds from 10 to 1100 mm min^−1^. The confocal images were tested on a widefield confocal microscope (Smartproof 5, Zeiss). The biodegradation of VegPU was done by immersing a piece of film in the lipase enzyme solution (1 mg mL^−1^ in 0.01 M PBS buffer with pH 7.4).

### The fabrication and testing of the ripening sensor on the soft gripper

The electrode pattern was designed in Auto CAD software and printed on a VegPU substrate by stencil printing with a metal stencil (thickness: 80 µm) and cured by sintering solution. Then, the printed sustainable EIS sensing electrodes were mounted on the soft pneumatic actuators (Rochu) with Ecoflex as the adhesive layer. After curing at 60 °C for 2 h, the smart soft gripper is ready for subsequent use. Tomatoes used for testing were purchased from the local market and the pressure for gripping the tomatoes purchased from a local market is 60 kPa. The EIS was measured by an electrochemical workstation (PGSTAT128N) from Metrohm Autolab. The impedance during shaking was tested by an LCR meter (Agilent E4980A) from Keysight Technologies. The storage of tomatoes is conducted in the ambient condition (20 °C, RH 30%). The weight, stiffness, and water-soluble content of tomatoes were measured by balance (ME 204, Mettler Toledo), fruit hardness tester (GY-3, Jingcheng Instrument), and refractometer (PAL-1, ATAGO), respectively.

### Reporting summary

Further information on research design is available in the Nature Portfolio Reporting Summary linked to this article.

### Supplementary information

Supplementary Information

Peer Review File

Reporting Summary

Description of Additional Supplementary Files

Supplementary Movie 1

Supplementary Movie 2

## Data Availability

The data that support the findings of this study are presented in the main article and the Supplementary Materials. Raw data necessary to reproduce the figures within this article are available in figshare database under 10.6084/m9.figshare.24270262.

## References

[1] Lv J, Thangavel G, Lee PS (2023). Reliability of printed stretchable electronics based on nano/micro materials for practical applications. Nanoscale.

[2] Yin L, Lv J, Wang J (2020). Structural innovations in printed, flexible, and stretchable electronics. Adv. Mater. Technol..

[3] Matsuhisa N, Chen X, Bao Z, Someya T (2019). Materials and structural designs of stretchable conductors. Chem. Soc. Rev..

[4] Kim DC, Shim HJ, Lee W, Koo JH, Kim D-H (2020). Material-based approaches for the fabrication of stretchable electronics. Adv. Mater..

[5] Gao D, Lv J, Lee PS (2022). Natural polymer in soft electronics: opportunities, challenges, and future prospects. Adv. Mater..

[6] Zhang C, Li Y, Chen R, Kessler MR (2014). Polyurethanes from solvent-free vegetable oil-based polyols. ACS Sustain. Chem. Eng..

[7] Zhang C (2021). Renewable castor-oil-based waterborne polyurethane networks: simultaneously showing high strength, self-healing, processability and tunable multishape memory. Angew. Chem. Int. Ed..

[8] Xia Y, Larock RC (2010). Castor oil-based thermosets with varied crosslink densities prepared by ring-opening metathesis polymerization (ROMP). Polymer.

[9] Wang Z (2012). Synthesis and characterization of novel soybean-oil-based elastomers with favorable processability and tunable properties. Macromolecules.

[10] Xia Y, Larock RC (2011). Castor-oil-based waterborne polyurethane dispersions cured with an aziridine-based crosslinker. Macromol. Mater. Eng..

[11] Lu Y, Larock RC (2008). Soybean-oil-based waterborne polyurethane dispersions: effects of polyol functionality and hard segment content on properties. Biomacromolecules.

[12] Kwon J (2022). Conductive ink with circular life cycle for printed electronics. Adv. Mater..

[13] Lee B (2023). Omnidirectional printing of elastic conductors for three-dimensional stretchable electronics. Nat. Electron..

[14] Cahn G (2020). The role of strain localization on the electrical behavior of flexible and stretchable screen printed silver inks on polymer substrates. Materialia.

[15] Kim B (2021). Rapid custom prototyping of soft poroelastic biosensor for simultaneous epicardial recording and imaging. Nat. Commun..

[16] Stier SP, Uhl D, Löbmann P, Böse H (2021). Dynamic electro-mechanical analysis of highly conductive particle-elastomer composites. J. Appl. Polym. Sci..

[17] Ma R, Tsukruk VV (2017). Seriography-guided reduction of graphene oxide biopapers for wearable sensory electronics. Adv. Funct. Mater..

[18] Shin SR (2016). A bioactive carbon nanotube-based ink for printing 2D and 3D flexible electronics. Adv. Mater..

[19] Lian M (2015). Gelatin-assisted fabrication of graphene-based nacre with high strength, toughness, and electrical conductivity. Carbon.

[20] Lee TH, Kim JH, Lee JY (2017). Fabrication of highly conductive fibers by metal ion-exchange using a simply modified wet-spinning process. Macromol. Res..

[21] Li Y (2017). Cellulose-nanofiber-enabled 3D printing of a carbon-nanotube microfiber network. Small Methods.

[22] Han WB (2023). Ultra-stretchable and biodegradable elastomers for soft, transient electronics. Nat. Commun..

[23] Kim K-S (2022). Biodegradable molybdenum/polybutylene adipate terephthalate conductive paste for flexible and stretchable transient electronics. Adv. Mater. Technol..

[24] Yang J (2020). Electrochemically active, compressible, and conducting silk fibroin hydrogels. Ind. Eng. Chem. Res..

[25] Jing X, Wang X-Y, Mi H-Y, Turng L-S (2019). Stretchable gelatin/silver nanowires composite hydrogels for detecting human motion. Mater. Lett..

[26] Hsiao L-Y (2020). Carbon nanotube-integrated conductive hydrogels as multifunctional robotic skin. Carbon.

[27] Thibodeau J, Ignaszak A (2020). Flexible electrode based on MWCNT embedded in a cross-linked acrylamide/alginate blend: conductivity vs. stretching. Polymers.

[28] Yan L (2021). Conductive cellulose bio-nanosheets assembled biostable hydrogel for reliable bioelectronics. Adv. Funct. Mater..

[29] Yao M, Su D, Wang W, Chen X, Shao Z (2018). Fabrication of air-stable and conductive silk fibroin gels. ACS Appl. Mater. Interfaces.

[30] Liu C, Zhang HJ, You X, Cui K, Wang X (2020). Electrically conductive tough gelatin hydrogel. Adv. Electron. Mater..

[31] Liu J (2020). Stretchable, self-healable, and reprocessable chemical cross-linked ionogels electrolytes based on gelatin for flexible supercapacitors. J. Mater. Sci..

[32] Song J (2020). Mechanically and electronically robust transparent organohydrogel fibers. Adv. Mater..

[33] Ye Y, Zhang Y, Chen Y, Han X, Jiang F (2020). Cellulose nanofibrils enhanced, strong, stretchable, freezing-tolerant ionic conductive organohydrogel for multi-functional sensors. Adv. Funct. Mater..

[34] Ling S (2018). Integration of stiff graphene and tough silk for the design and fabrication of versatile electronic materials. Adv. Funct. Mater..

[35] López Barreiro D (2019). Conductive silk-based composites using biobased carbon materials. Adv. Mater..

[36] Zeng Y (2022). Highly stretchable fatty acid chain-dangled thermoplastic polyurethane elastomers enabled by h-bonds and molecular chain entanglements. ACS Sustain. Chem. Eng.

[37] Ying WB (2021). A biologically muscle-inspired polyurethane with super-tough, thermal reparable and self-healing capabilities for stretchable electronics. Adv. Funct. Mater..

[38] Parida K (2019). Extremely stretchable and self-healing conductor based on thermoplastic elastomer for all-three-dimensional printed triboelectric nanogenerator. Nat. Commun..

[39] Liu Z (2019). Biomimetic materials with multiple protective functionalities. Adv. Funct. Mater..

[40] Fu D (2018). A facile dynamic crosslinked healable poly(oxime-urethane) elastomer with high elastic recovery and recyclability. J. Mater. Chem. A.

[41] Park S-J, Jin F-L, Lee J-R (2004). Synthesis and thermal properties of epoxidized vegetable oil. Macromol. Rapid Commun..

[42] Mustata F, Tudorachi N, Rosu D (2011). Curing and thermal behavior of resin matrix for composites based on epoxidized soybean oil/diglycidyl ether of bisphenol A. Compos. B Eng..

[43] Yan C (2021). Semiquantitative solid-state NMR study of the adsorption of soybean oils on silica and its significance for rubber processing. Langmuir.

[44] Caillol S (2012). Synthesis of new polyester polyols from epoxidized vegetable oils and biobased acids. Eur. J. Lipid Sci. Technol..

[45] Xiong J, Thangavel G, Wang J, Zhou X, Lee PS (2020). Self-healable sticky porous elastomer for gas-solid interacted power generation. Sci. Adv..

[46] Zhang L (2019). A highly efficient self-healing elastomer with unprecedented mechanical properties. Adv. Mater..

[47] Kim J, Kumar R, Bandodkar AJ, Wang J (2017). Advanced materials for printed wearable electrochemical devices: a review. Adv. Electron. Mater..

[48] Guillen GR, Pan Y, Li M, Hoek EMV (2011). Preparation and characterization of membranes formed by nonsolvent induced phase separation: a review. Ind. Eng. Chem. Res..

[49] Lv J (2021). Printable elastomeric electrodes with sweat-enhanced conductivity for wearables. Sci. Adv..

[50] Acik G, Kamaci M, Altinkok C, Karabulut HRF, Tasdelen MA (2018). Synthesis and properties of soybean oil-based biodegradable polyurethane films. Prog. Org. Coat..

[51] Petrović ZS, Xu Y, Milić J, Glenn G, Klamczynski A (2010). Biodegradation of thermoplastic polyurethanes from vegetable oils. J. Polym. Environ..

[52] Kim B (2021). Rapid custom prototyping of soft poroelastic biosensor for simultaneous epicardial recording and imaging. Nat. Commun..

[53] Vásquez Quintero A, Verplancke R, De Smet H, Vanfleteren J (2017). Stretchable electronic platform for soft and smart contact lens applications. Adv. Mater. Technol..

[54] Lee S (2020). Making something out of nothing: enhanced flaw tolerance and rupture resistance in elastomer–void “negative” composites. Extreme Mech. Lett..

[55] Kim B-J (2012). Fatigue-free, electrically reliable copper electrode with nanohole array. Small.

[56] Zhang X-W, Pan Y, Zheng Q, Yi X-S (2000). Time dependence of piezoresistance for the conductor-filled polymer composites. J. Polym. Sci. B Polym. Phys..

[57] Ohm Y (2021). An electrically conductive silver–polyacrylamide–alginate hydrogel composite for soft electronics. Nat. Electron..

[58] Li W (2019). Three-dimensional stretchable and transparent conductors with controllable strain-distribution based on template-assisted transfer printing. ACS Appl. Mater. Interfaces.

[59] Matsuhisa N (2017). Printable elastic conductors by in situ formation of silver nanoparticles from silver flakes. Nat. Mater..

[60] Valentine AD (2017). Hybrid 3D printing of soft electronics. Adv. Mater..

[61] Nam HJ, Kang SY, Park JY, Choa S-H (2019). Intense pulse light sintering of an Ag microparticle-based, highly stretchable, and conductive electrode. Microelectron. Eng..

[62] Guo W (2019). Matrix-independent highly conductive composites for electrodes and interconnects in stretchable electronics. ACS Appl. Mater. Interfaces.

[63] Kim SH (2019). An ultrastretchable and self-healable nanocomposite conductor enabled by autonomously percolative electrical pathways. ACS Nano.

[64] Oh Y (2017). Selective photonic sintering of Ag flakes embedded in silicone elastomers to fabricate stretchable conductors. J. Mater. Chem. C.

[65] Pan C (2019). Silver-coated poly(dimethylsiloxane) beads for soft, stretchable, and thermally stable conductive elastomer composites. ACS Appl. Mater. Interfaces.

[66] Wang T, Liu Q, Liu H, Xu B, Xu H (2022). Printable and highly stretchable viscoelastic conductors with kinematically reconstructed conductive pathways. Adv. Mater..

[67] El Khaled D, Castellano NN, Gazquez JA, García Salvador RM, Manzano-Agugliaro F (2017). Cleaner quality control system using bioimpedance methods: a review for fruits and vegetables. J. Clean. Prod..

[68] Chowdhury, A., Bera, T. K., Ghoshal, D. & Chakraborty, B. Studying the electrical impedance variations in banana ripening using electrical impedance spectroscopy (EIS). In *Proc. 2015 Third International Conference on Computer, Communication, Control and Information Technology (C3IT)* 1–4 (IEEE, 2015).

[69] Li J, Xu Y, Zhu W, Wei X, Sun H (2019). Maturity assessment of tomato fruit based on electrical impedance spectroscopy. Int. J. Agric. Biol. Eng..

[70] Figueiredo Neto A, Cárdenas Olivier N, Rabelo Cordeiro E, Pequeno de Oliveira H (2017). Determination of mango ripening degree by electrical impedance spectroscopy. Comput. Electron. Agric..

[71] Ibba P (2021). Supervised binary classification methods for strawberry ripeness discrimination from bioimpedance data. Sci. Rep..

[72] Islam M, Wahid K, Dinh A (2018). Assessment of ripening degree of avocado by electrical impedance spectroscopy and support vector machine. J. Food Qual..

[73] Yovcheva T (2013). Investigation of apples aging by electric impedance spectroscopy. Bulg. Chem. Commun..

[74] Saleh MA, Soliman M, Mousa MA, Elsamanty M, Radwan AG (2020). Design and implementation of variable inclined air pillow soft pneumatic actuator suitable for bioimpedance applications. Sens. Actuators A Phys..

[75] Zhang B, Xie Y, Zhou J, Wang K, Zhang Z (2020). State-of-the-art robotic grippers, grasping and control strategies, as well as their applications in agricultural robots: a review. Comput. Electron. Agric..

[76] Beckles DM (2012). Factors affecting the postharvest soluble solids and sugar content of tomato (*Solanum lycopersicum* L.) fruit. Postharvest Biol. Technol..

[77] Goulao LF, Oliveira CM (2008). Cell wall modifications during fruit ripening: when a fruit is not the fruit. Trends Food Sci. Technol..

