# Supplementary material for: Printed sustainable elastomeric conductor for soft electronics
Source: Nat Commun. 2023 Nov 6;14:7132. doi: 10.1038/s41467-023-42838-7 (PMC10628110; doi:10.1038/s41467-023-42838-7)
Supplement: Supplementary file 1 — Supplementary Information [file 41467_2023_42838_MOESM1_ESM.pdf]

Printed porous sustainable elastomeric conductor for soft electronics

Jian Lv[1,2,3+], Gurunathan Thangavel[1,4+], Yangyang Xin[1,2], Dace Gao[1], Wei Church Poh[1], Shaohua Chen[1] and Pooi See Lee[1,2*]

1. School of Materials Science and Engineering, Nanyang Technological University, 50 Nanyang Avenue, Singapore, 639798

2. Singapore-HUJ Alliance for Research and Enterprise (SHARE), Smart Grippers for Soft Robotics (SGSR), Campus for Research Excellence and Technological Enterprise, Singapore 138602, Singapore

3. Present address: Frontier Institute of Science and Technology, Xi'an Jiaotong University, Xi'an, 710049 China

4. Present address: Advanced Materials Research Center, Technology Innovation Institute (TII), Masdar City, Abu Dhabi, P.O. Box 9639, United Arab Emirates

[+]These authors contributed equally to this work

[*]Corresponding author. Email: pslee@ntu.edu.sg

This PDF file includes:

Supplementary Table 1-2

Supplementary Fig. 1-49

References

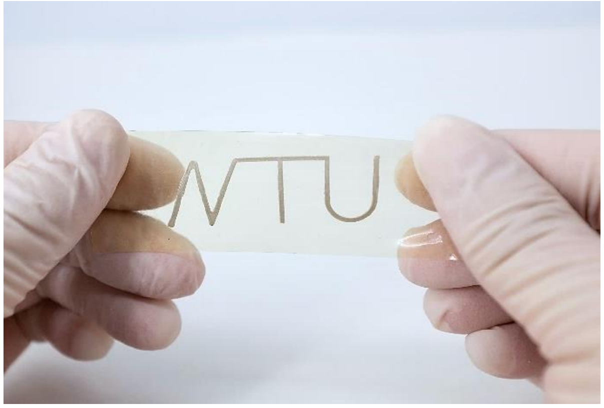

Supplementary Fig. 1 | The photo image of one printed VegPU/Ag pattern on the VegPU substrate. The VegPU/Ag ink was screen-printed on a VegPU thin film and then cured by sintering solution.

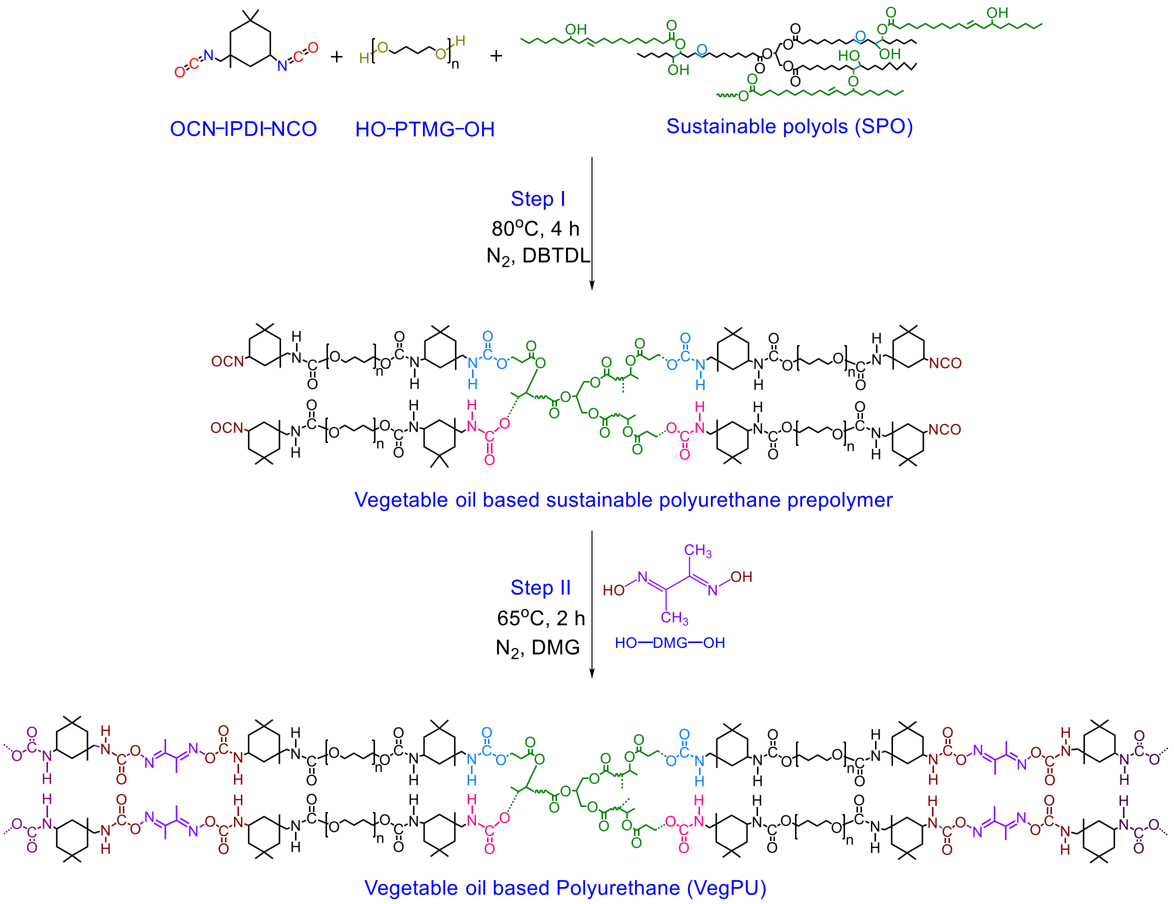

Supplementary Fig. 2 | Synthesis of representative VegPU from sustainable polyols. A series of VegPU were synthesized from SPO and PTMG with IPDI at a molar ratio of 0.8:0.5:1.5 (-OH/-NCO). First, the PTMG-IPDI-based NCO-terminated precursor was prepared by the PTMEG and IPDI chemical reaction with a 2: 3 mole ratio at 75 -80 °C under a nitrogen condition for 6 hours. The PTMEG-IPDI precursor (4 mmol) and SPO (8.03 mmol) were solubilized in anhydrous DMF (20 ml) and were left to react for 4 hours at 80 °C. The reacting solution was then given one drop of DBTDL and stirred for another 30 minutes. Subsequently, dimethylglyoxime (DMG) (4.04 mmol) as a chain extender was diluted in DMF (5mL), and the resulting solution was added into the reaction system that was further maintained at 65 °C for 2 hours. The resultant polymer solution was then placed into a glass mold (length × width: 100 mm × 50 mm) and dried in an 80 °C oven for 12 hours.

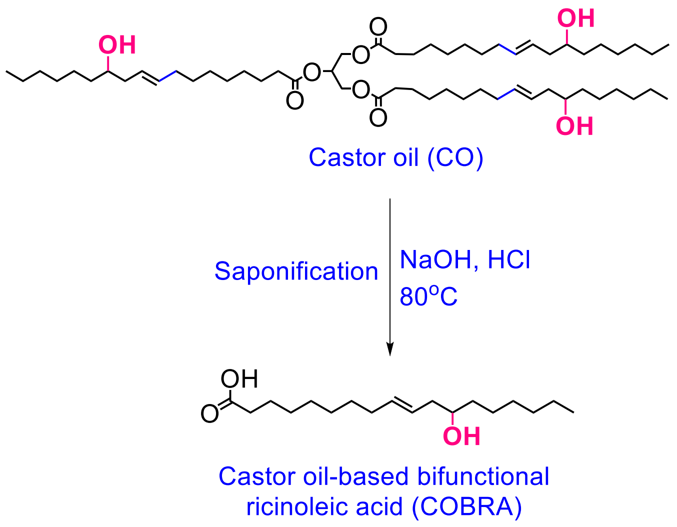

Supplementary Fig. 3 | Synthesis of castor oil-based bifunctional ricinoleic acid. Bifunctional ricinoleic acid could be acquired through either saponification or fractional distillation of castor oil that has been hydrogenated. Initially, required quantities of castor oil were saponified into bifunctional ricinoleic acid by heating a sodium hydroxide (NaOH) mixture at 80-85 °C for 4 hours. The COBRA solution was then neutralized using diluted hydrochloric acid (HCl). The organic phase has been filtered after neutralization by washing with double-distilled water (*dd*H_2O). The dissolution-decantation process was routine four times, and the product was dried over magnesium sulfate ($MgSO_4$). The majority of the bifunctional fatty acids in castor oil are ricinoleic acid (88 to 91%), and additional acids are present, including linolenic, linoleic, and oleic acids.

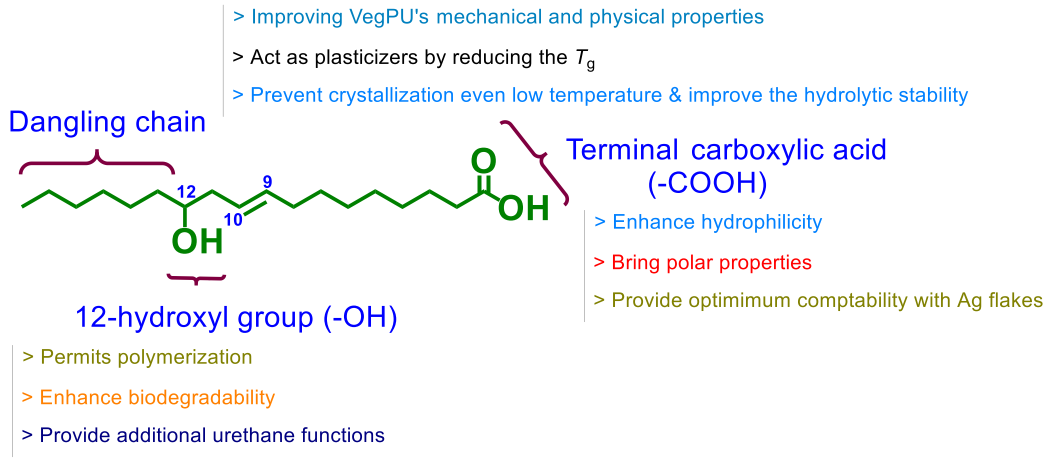

Supplementary Fig. 4 | The importance and advantages of bifunctional ricinoleic acid functional units of dangling chain, -COOH and -OH in the VegPU preparations. Ricinoleic acid (12 hydroxy–cis–9–octadecenoic acid) is the major hydroxylated aliphatic unsaturated fatty acid in castor oil (>90%) and a key substrate for the polymerization of precursors to produce sustainable VegPU.

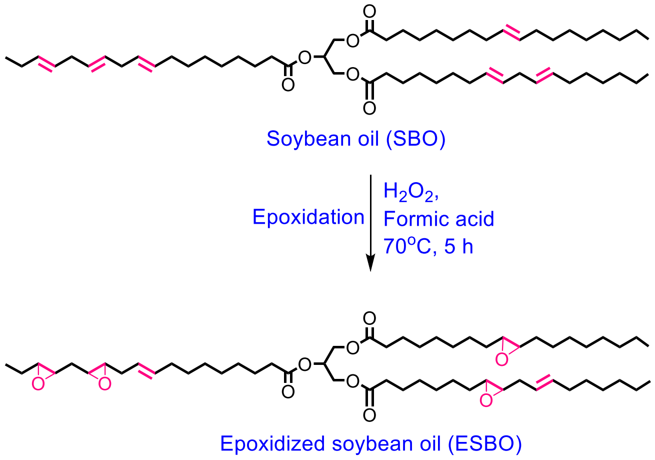

Supplementary Fig. 5 | Synthesis of epoxidized SBOs. In a typical experiment, a measured quantity of soybean oil (0.5038 mol) was processed at 70 °C for 1 hour in a 250 mL round-bottle flask with a thermometer, and an oil bath. Thereafter, a mixture of hydrogen peroxide (H_2O_2, 0.2855 mol) and formic acid (HCOOH, 0.0570 mol) was introduced to the soybean oil with the catalyst at a syringe pump rate of 0.20 cm^3 min^{-1}. The resultant mixture was stirred periodically and kept at the same temperature of 70 °C for 5 hours. After the addition of the oxidant mixture, the reaction proceeded for the preferred duration of time.

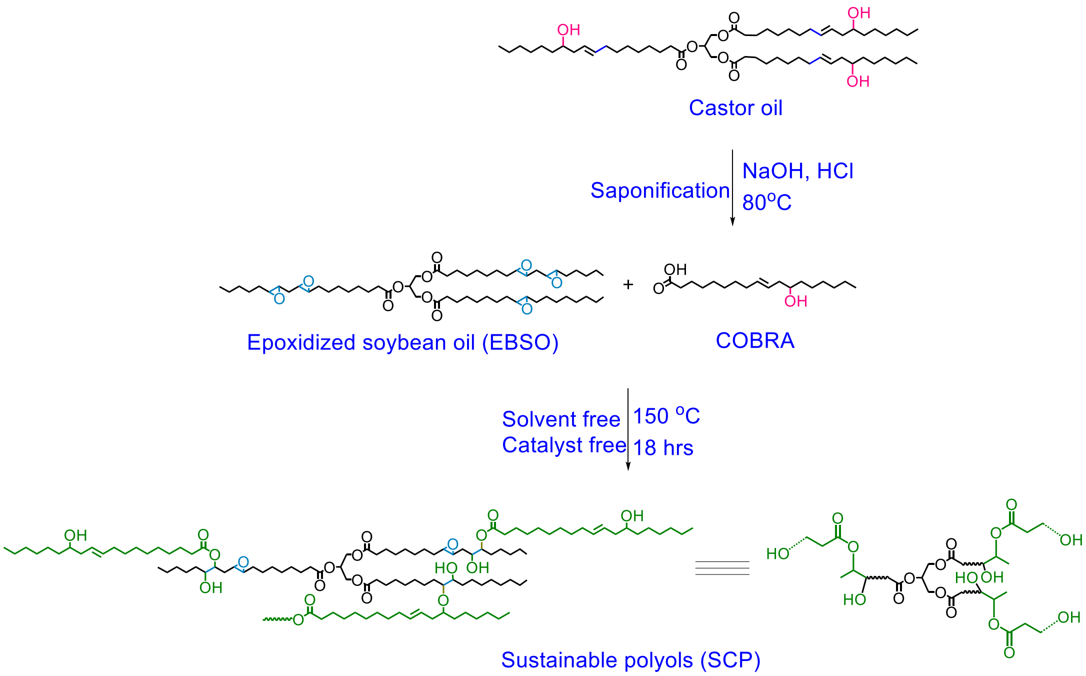

Supplementary Fig. 6 | Synthesis of sustainable polyols. Firstly, COBRA was placed in a flask with a mole ratio of the carboxyl group of 1.5, stirred with a mechanical stirrer, and kept at 150 °C in an N_2-free environment. Then, ESBO at a mole ratio of 3 to the epoxy group was added drop by drop while vigorously stirring. After being mixed fully, the mixture was kept at 140 to 180 °C overnight with the constant stirring condition. To quench the reaction, 30% ammonia in water was added to the solution mixture. After the reaction was complete, the finished SPO products were obtained with Et_2O and washed at least five times with double-distilled water. After drying the precipitate with $MgSO_4$, it was filtered. After removing the organic solvent with Rotavapor and vacuum, the clear viscous pale yellow SPO was obtained.

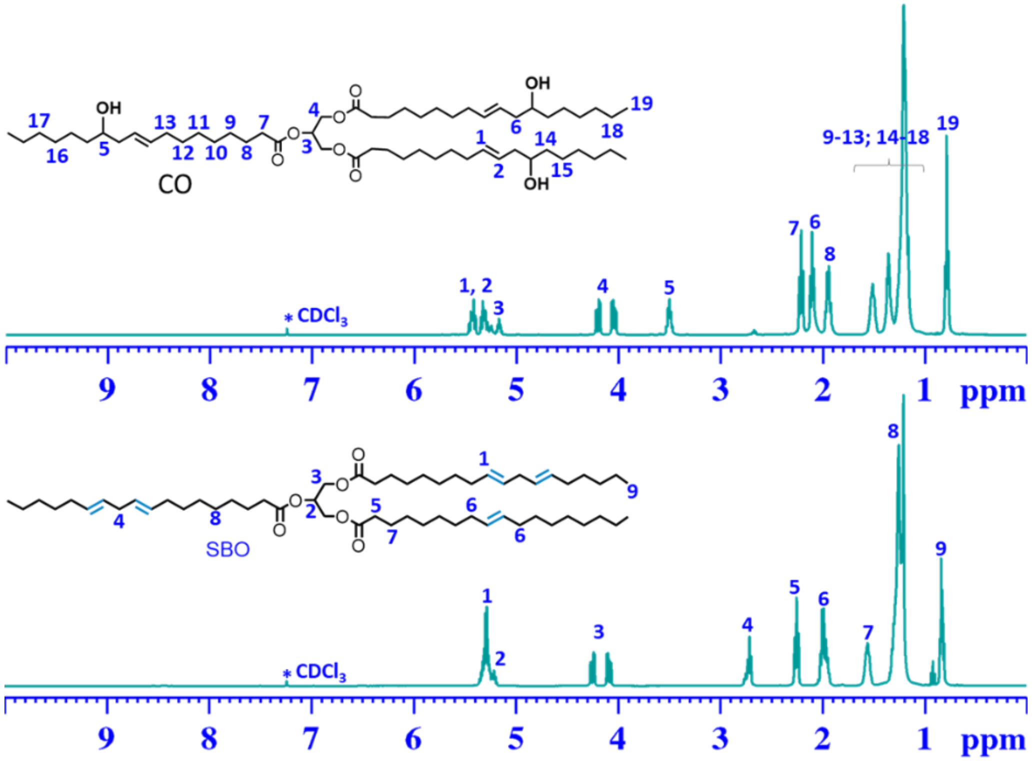

Supplementary Fig. 7 | ^{1}H-NMR spectra of castor oil (CO) and soybean oil (SBO). The 3° proton of the -$CH_2C\mathbf{H}CH_2$- the backbone of castor oil is at 5.1–5.4 ppm, the -CH_2 proton of the -$C\mathbf{H}_2$-$CHC\mathbf{H}_2$- backbone is at 4.2–4.3 ppm, and the 3° hydrogen neighboring to the -OH proton in the ricinoleic acid chain is at 3.4–3.7 ppm. The SBO spectrum demonstrates that each triacylglycerol forms five double bonds (-C=C-).

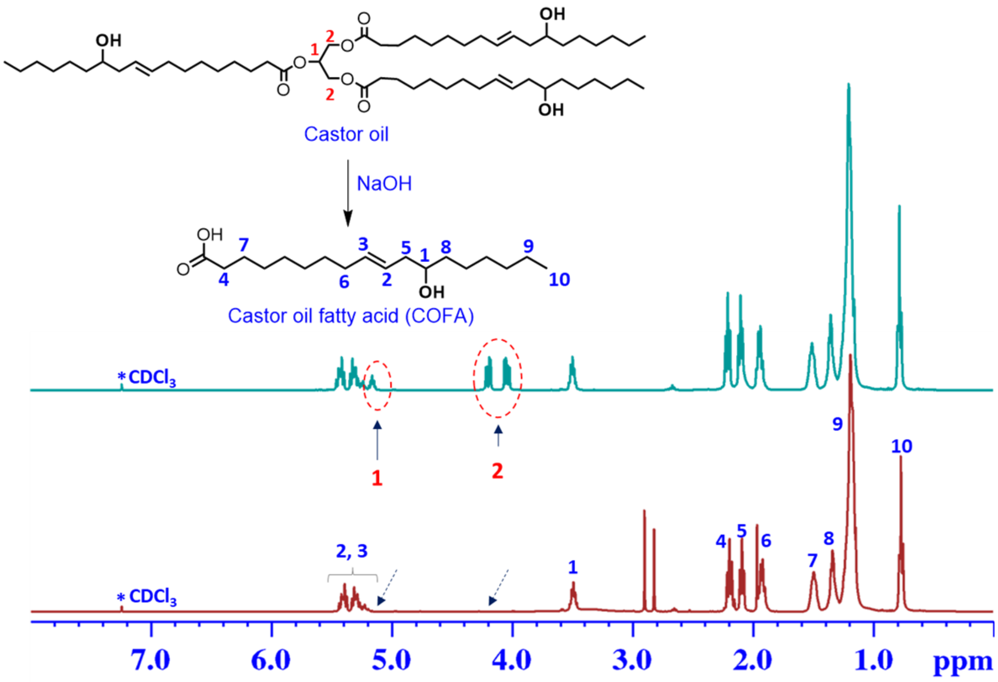

Supplementary Fig. 8 | ^{1}H-NMR spectra of CO and COBRA. The 3° proton of the -$CH_2C\mathbf{H}CH_2$- the backbone of castor oil is at 5.1–5.4 ppm, the -CH_2 proton of the -$C\mathbf{H}_2$-$CHC\mathbf{H}_2$- backbone is at 4.2–4.3 ppm, and the 3° hydrogen neighboring to the -OH proton in the ricinoleic acid chain is at 3.4–3.7 ppm. The peaks at 5.1–5.4 ppm and 4.2–4.3 ppm went away after the reduction reaction. On the other hand, the peak intensity at 3.4–3.7 ppm went up, which shows that triglyceride was completely broken down, which led to the formation of primary hydroxyl groups (-OH). It is therefore concluded that COBFA was successfully prepared.

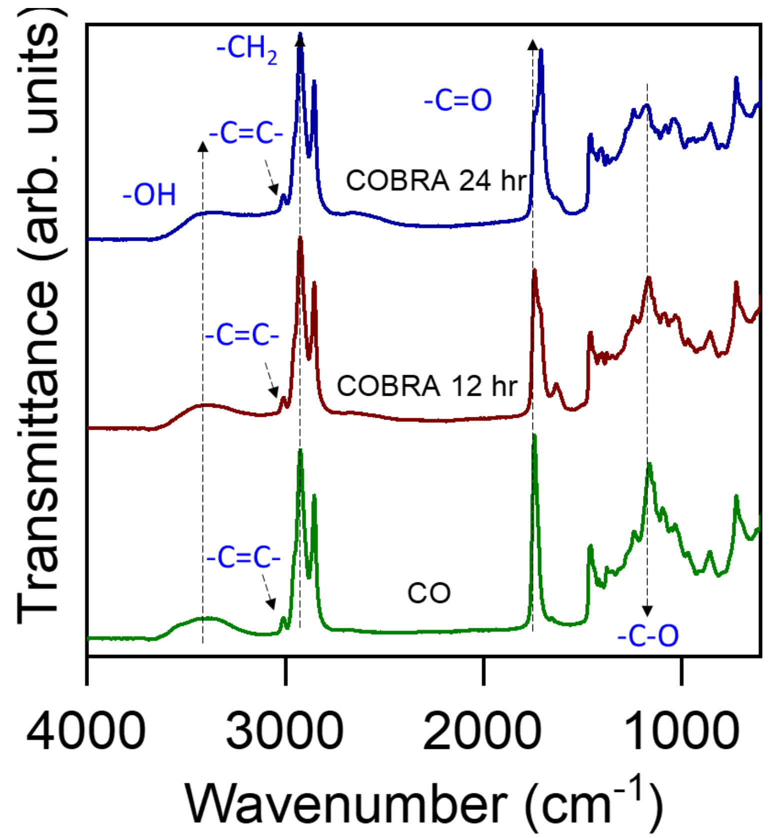

Supplementary Fig. 9 | FTIR spectra of CO and COBRA at various reaction times. The broad absorption band at around 3400^{-1} and 1750^{-1} can be assigned to hydroxyl and carbonyl groups, respectively.

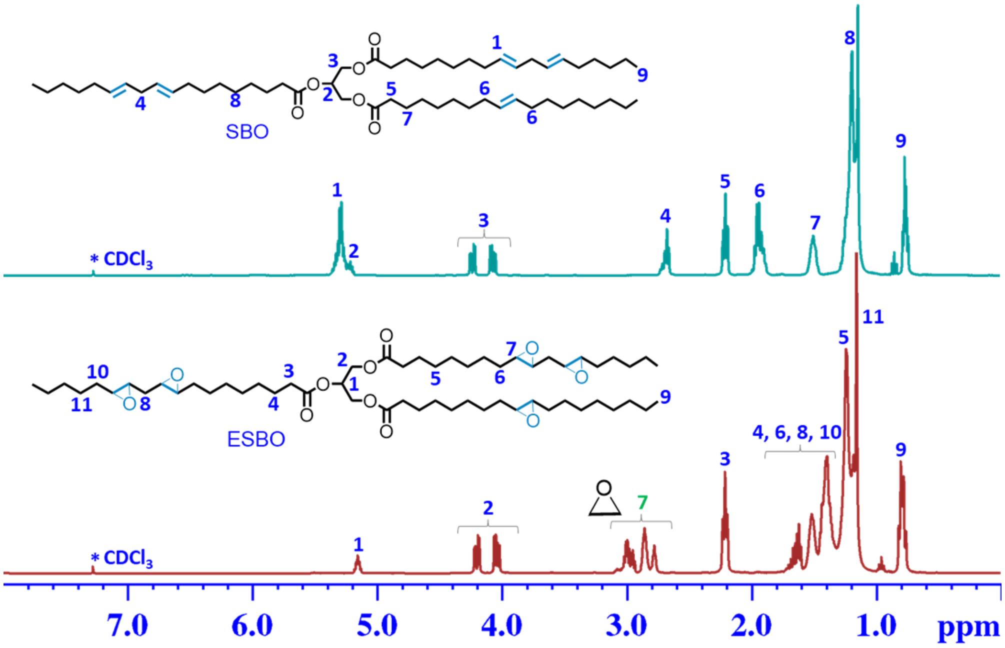

Supplementary Fig. 10 | ^{1}H-NMR spectra of soybean oil (SBO) and epoxidized soybean oil (ESBO). The SBO spectrum demonstrates that each triacylglycerol forms five double bonds (-C=C-). The ESBO spectrum illustrates that unsaturated fatty acids were transformed into epoxy groups labeled as diepoxides and monoepoxides, with signals having appeared at 3.06–3.16 ppm (-CHOC**H**-CH_2-C**H**OCH-) and 2.5–3.1 ppm (-C**H**OCH-CH_2-CHOC**H**-). The peak at 1.45–1.50 ppm methylene protons (-CH_2) neighboring epoxy group (-CH_2-C**H**$_2$-CHOCH-) and at 2.3 ppm α-methylene group (**α**-CH_2) to acyl group (CH_2-C**H**$_2$-C=O-O-).

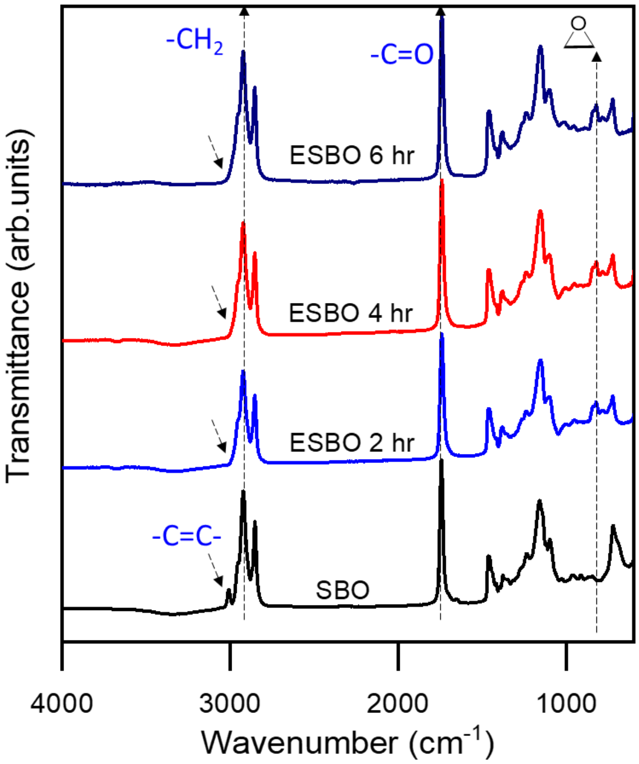

Supplementary Fig. 11 | FTIR spectra of SBO and epoxidized SBO (ESBO) at various reaction times. The characteristic absorption bands at 822, 1173, 1725, and 2913 cm $^{-1}$ are attributable to the stretching vibration of epoxy, C–O, C=O, and C-H groups of the ESBO, respectively. The epoxidation of epoxidized SBOs (ESBO) was followed by the disappearance of the C = C peak at 3011 cm-1 and the appearance of the new broad epoxy peak stretching vibration between 862-809 cm $^{-1}$.

1

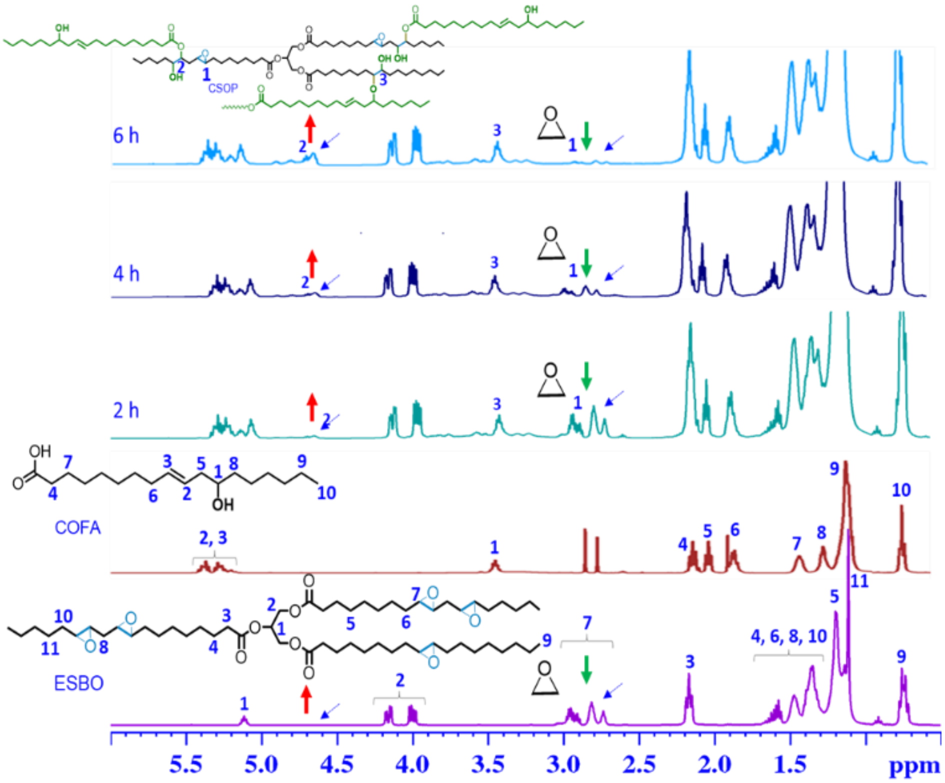

Supplementary Fig. 12 | ^{1}H-NMR spectra of sustainable polyols (SPO) for distinct intervals of carboxyl to epoxy group reactions. As reaction times increased, the peaks at 2.8-3.2 ppm, representing the epoxy groups, decreased. The peaks at 2.7-3.1 ppm representing epoxy groups, decreased as reaction times increased. On the other hand, new peaks corresponding 3° to Hto atoms next to the newly formed ester groups were detected between 4.5-4.9 ppm. The peaks showing where hydrogen is attached to carbons near the ester overlap with those where hydrogen is attached to carbons near the hydroxyl.

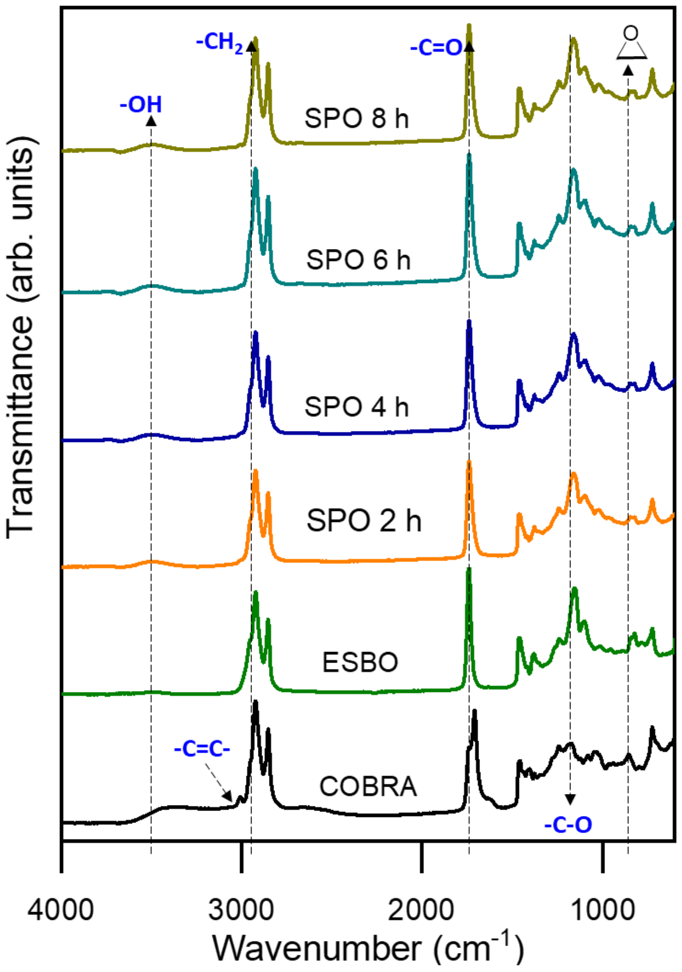

Supplementary Fig. 13 | FTIR spectra of COBRA, ESBO, and SPO at various reaction times intervals. The oxirane absorption bands at 822 cm^{-1}, which represent epoxy groups, appeared, while the carbon-carbon (-C=C-) double bonds at 3001-3012 cm^{-1} almost vanished. The absence of epoxy groups in polyols indicated that the epoxy groups in ESBO had been ring-opened. The epoxy groups in epoxidized SBO decreased after the ring-opening reaction was initiated by COBRA, while a broad peak at 3395 cm^{-1} appears, indicating that the epoxy groups in ESBO were ring-opened and sustainable hydroxyl (-OH) polyols were successfully formed.

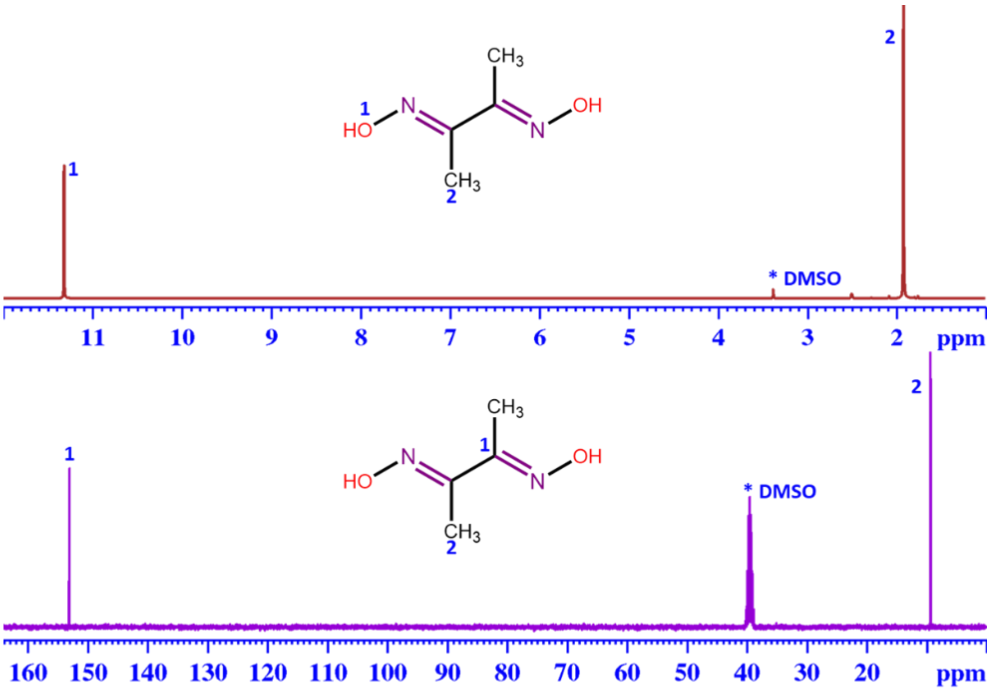

Supplementary Fig. 14 | ^{1}H and ^{13}C-NMR spectra of dimethylglyoxime (DMG). The dimethylglyoxime was purchased from Sigma-Aldrich without any purification.

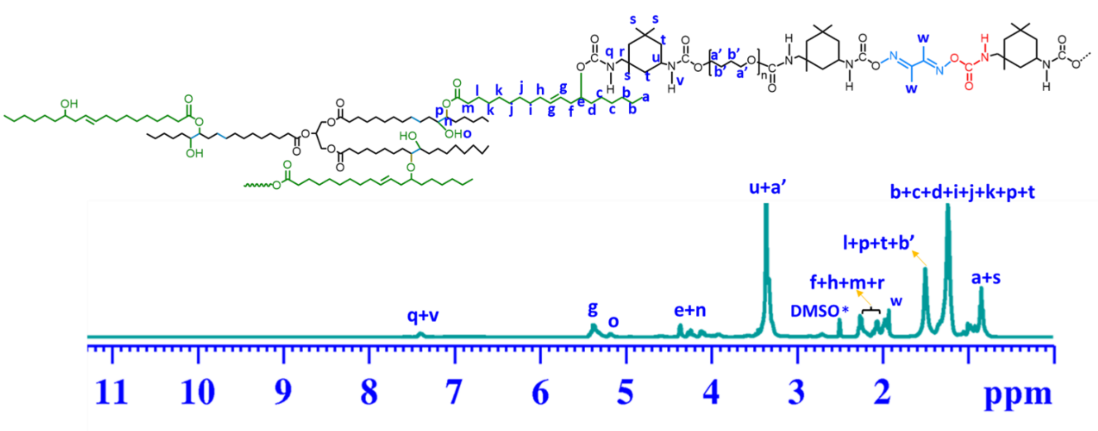

Supplementary Fig. 15 | ^{1}H-NMR spectra of VegPU. ^{1}H-NMR (400 MHz, DMSO, 25 °C) δ (ppm): δ 7.23-7.38 (−C(O)$\underline{\text{NH}}$−), δ 5.14 (-C$\underline{\text{H}}$=C$\underline{\text{H}}$-), δ 5.46 (−O$\underline{\text{H}}$), δ 4.21−4.35 (−O−C$\underline{\text{H}}$−), δ 3.32 (CO−NH−$\underline{\text{CH}}$−), δ 3.28 (CO−O−$\underline{\text{CH}_2}$), δ 2.17−2.30 (−$\underline{\text{CH}_2}$−), δ 2.01 (6H, CH=$\underline{\text{C}}$−$\underline{\text{CH}_3}$), δ 1.52-1.68 (−C−$\underline{\text{CH}_2}$−), δ 1.18-1.50 (−$\underline{\text{CH}_2}$−$\underline{\text{CH}_2}$−$\underline{\text{CH}_3}$), and δ 0.81-0.93 (CH$_2$−$\underline{\text{CH}_3}$).

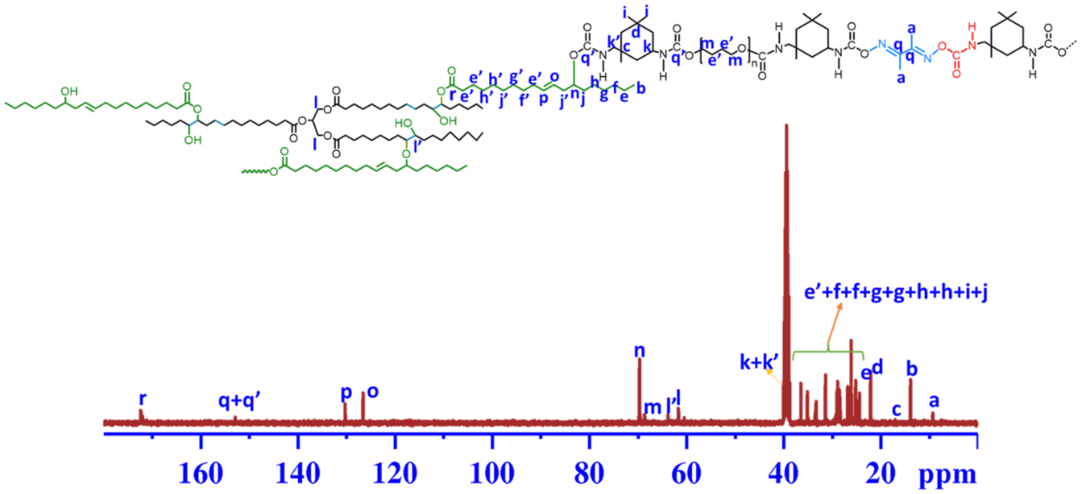

Supplementary Fig. 16 | ^{13}C-NMR spectra of VegPU. ^{3}C-NMR (400 MHz, DMSO, 25 ^{o}C) δ (ppm): 173.2 (-CH$_2$-CO-O-), 154.1-155.6 (-NH-CO-O-), 131.3 (-C=C), 126.8 (-C=C), 71.6 (CO-O-C), 68.2 (CH$_2$-O-), 61.8-64.4 (HO-C), 40.9-42.1 (NH-C), 8.3-38.4 (CH$_3$-CH$_2$ and CO-CH$_2$, etc).

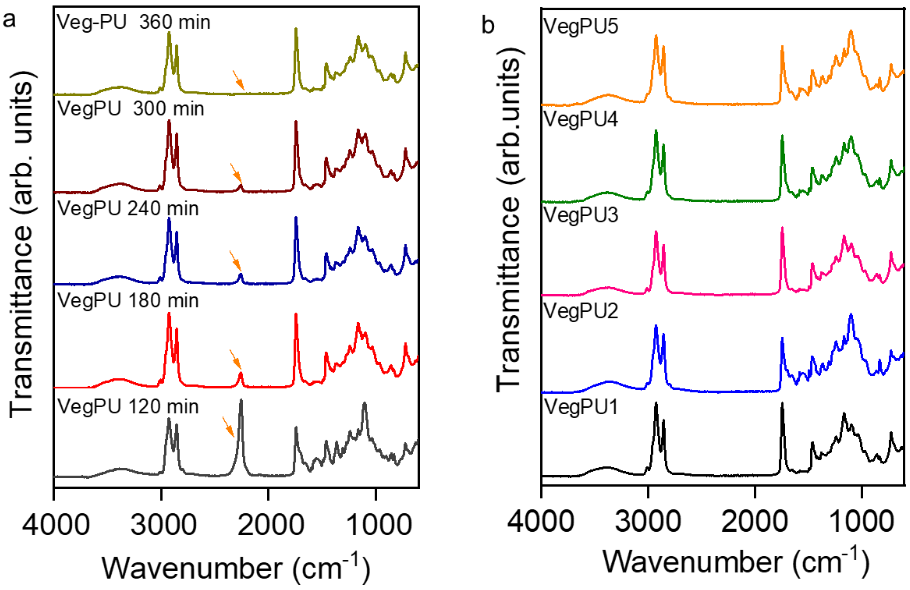

Supplementary Fig. 17 | FTIR spectra of a sustainable VegPU prepolymer: at various time intervals (a) and different ratios of oxime (b). The peak at 2268 cm^{-1} was designated to the NCO groups of VegPU at fixed temperature and variable time periods. The strength of the NCO peak at 2268 cm^{-1} reduces over time progressively with specific temperature, confirming the reaction between the -NCO group of IPDI and the -OH groups of PTMG, SCP, and DMG. After 360 minutes, there was almost no NCO prepolymer band at 2268 cm^{-1}, indicating that the NCO and the -OH bonds of PTMG, SCP, and DMG had fully reacted and the reaction was complete, with no reactants remaining. B) The following characteristic bands were found in the VegPU result: 3340-3356 cm^{-1} (N-H amide stretching), 3001-2800 cm^{-1} (symmetric and anti-symmetric aliphatic stretching), 1725 cm^{-1} (C=O carbonyl stretching), 1453 cm^{-1} (C-H), and 1539 cm^{-1} (C-NH bending). The bands at 991 cm^{-1} and 944 cm^{-1} were assigned to the out-of-plane C-H and C=C bending vibrations. The peak representing the N-O stretching vibration of oxime appears at 975-988 cm^{-1}.

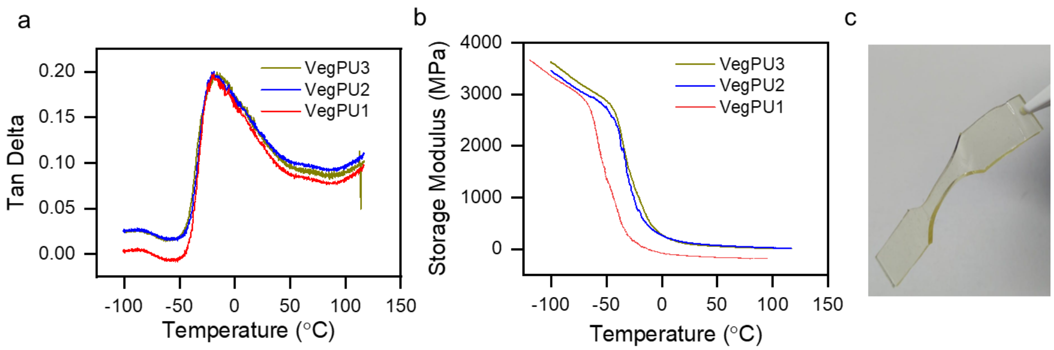

Supplementary Fig. 18 | The DMA of VegPU. The loss factor (a) and storage modulus (b) as functions of temperature for VegPU films measured by DMA from -100 °C to 120 °C under a room temperature, at a heating rate of 3 °C min^{-1} and a frequency of 1 Hz. (c) the image of the VegPU testing specimen.

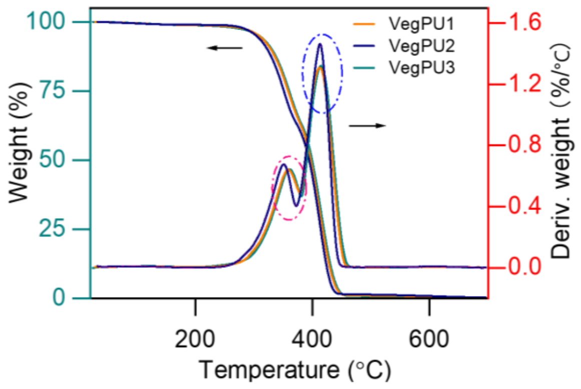

Supplementary Fig. 19 | TGA weight loss and derivative curves for the VegPU films. The first degradation of the VegPUs observed at temperatures between 240-350 ℃ was attributed to the decomposition of labile carbamate-oxime groups. The second degradation observed in the temperature range from 350-500 ℃ resulted from chain scission in the sustainable polyols.

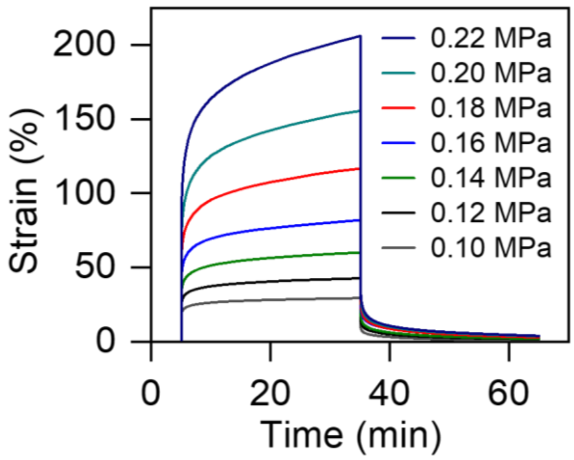

Supplementary Fig. 20 | The creep and recovery strain-time curve of VegPU under different applied stress at 25 ℃. The creep stress varied from 0.10 to 0.22 MPa. When the stress was increased from 0.10 to 0.22 MPa, the strain increased by 28 to 220%. The elastic response of the VegPU films causes an instantaneous increment in strain in all VegPU creep curves. At the end of the applied load period, viscous flow is observed. When the stress is removed, the strain rapidly decreases to the initial elastic response. During the rest period, the polymer makes an attempt to recapture its original shape through time-dependent molecular stress relief.

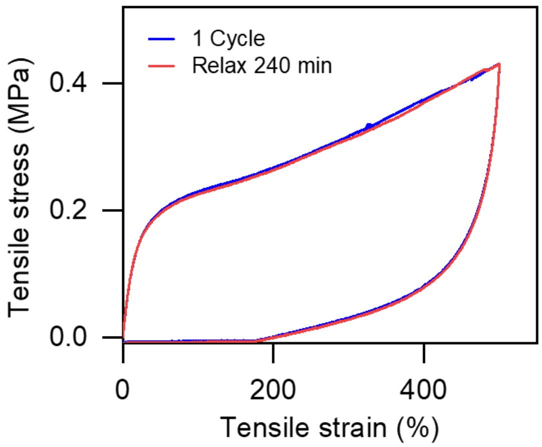

Supplementary Fig. 21 | The cyclic tensile (500%) tests of VegPU for the first cycles and after 240 min relaxation. Strain rate: 100 mm/min; width of sample: 14 mm; thickness: 1 mm; length of gauge: 40 mm.

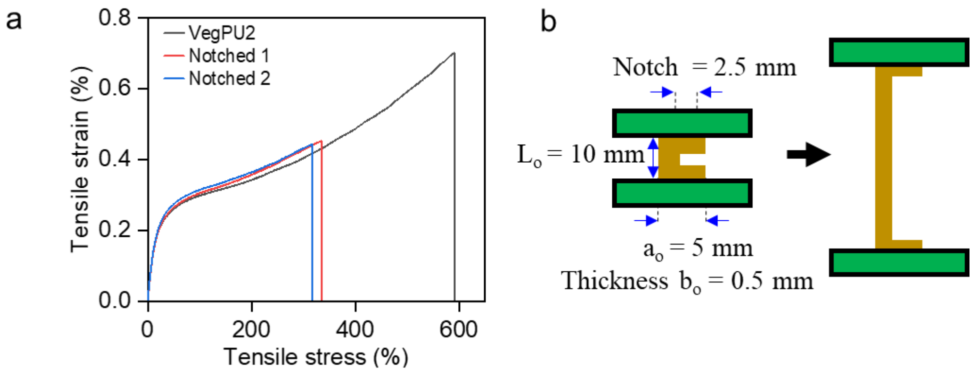

Supplementary Fig. 22 | The notch testing for VegPU. The stress-extension curves of unnotched VegPU and notched VegPU film (A); (B) the size of the notched area. 2 mm thickness, 10 mm length, and 5 mm width was notched along a single edge at a length of 1 mm, and the film was extended at a loading rate of 100 mm/min.

Supplementary Table 1 | The comparison of VegPU with other petrol-based PU elastomers and sustainable PU elastomers. HPUA: Hydrophilic poly(urethane-acrylate) elastomer; PDPU: polysiloxane-dimethylglyoxime–based polyurethane: poly(urethane-acrylate) elastomer; PU-OOP: polyurethane-olive oil polyol; PU-MSO: polyurethane- soybean oil-based polyols; POPU: palm oil polyurethane.

Sample	Sustainable polyols	Sustainable percentage (%)	Tensile strength (MPa)	Elongation at break (%)	Reference
HPUA	None	0	3.69	4954.83	[2]
PDPU	None	0	0.9	1800	[3]
PUA	None	0	7	5000	[4]
PU-OOP	Olive oil	70.25%	4.7 ±0.4	331.5±12.9	[5]
PU-MSO	Soybean oil	10.71	11.60±0.24	610±20	[6]
POPU	Palm oil	34.23	1.17±0.4	608.52	[7]
VegPU	Castor and soybean oil	75-81%	0.66	797	Our work

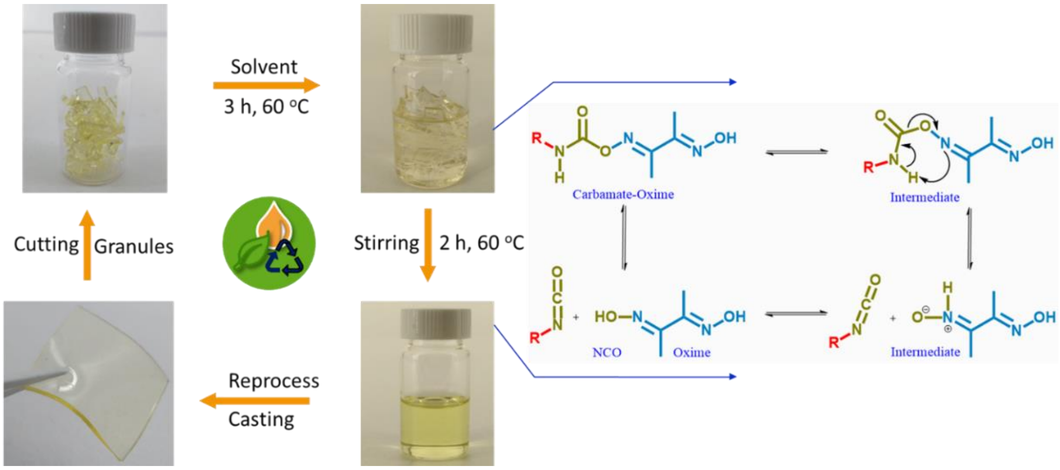

Supplementary Fig. 23 | VegPU being recycled through the process of solution casting. After cutting the fresh VegPU film into granules, the granules were dissolved in the DMF solvent and stirred for 5 h at 60 ℃ to produce a homogenous solution. Afterward, the solution was poured into the glass mold and allowed to dry at a temperature of 80 ℃ for a period of 12 h.

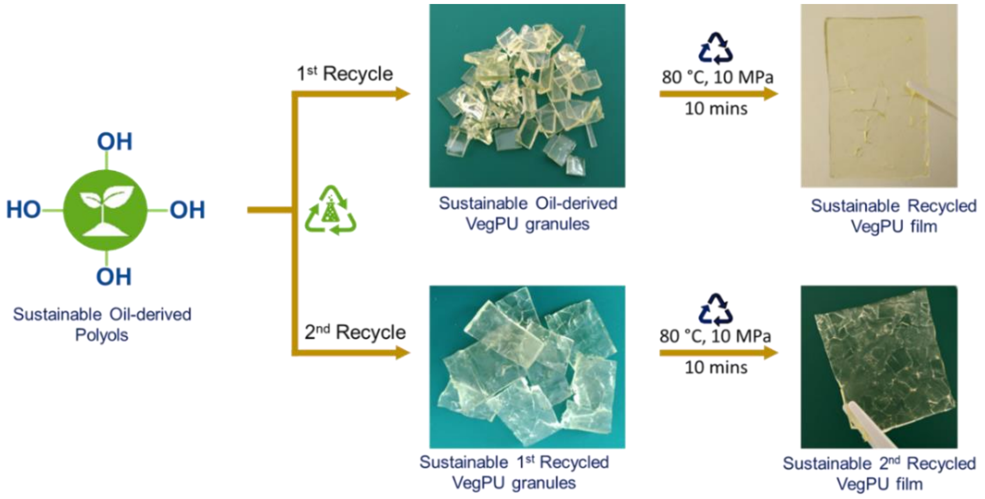

Supplementary Fig. 24 | VegPU being recycled through the process of hot-pressing (Condition: 80 ℃, 10 MPa, 10 mins). The VegPU film was cut into small pieces and hot pressed for 10 minutes at 80 ℃ with a pressure of 10 MPa.

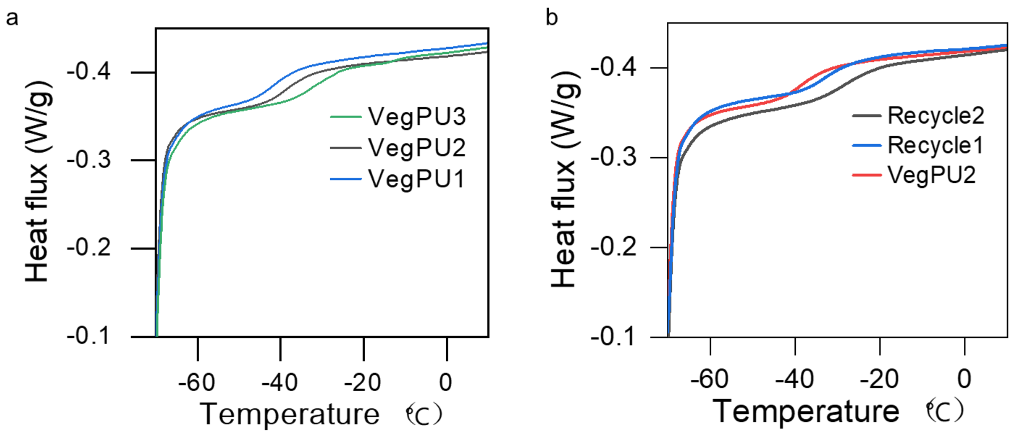

Supplementary Fig. 25 | DSC thermograms (a) of the different VegPU films and recycling curves (b) of VegPU2 (Recycle 1: solution casting and Recycle 2: hot-pressing). The DSC measurements were carried out at heating rates of 10 ℃ /min in a nitrogen-free environment. The absence of melting or crystallization transitions in the DSC curves indicates that these VegPUs are amorphous. Each curve has one Tg, which corresponds to the urethane segments of the obtained VegPU. The three VegPUs Tg were remarkably similar, which can be attributed to the urethane and hydroxyl functions.

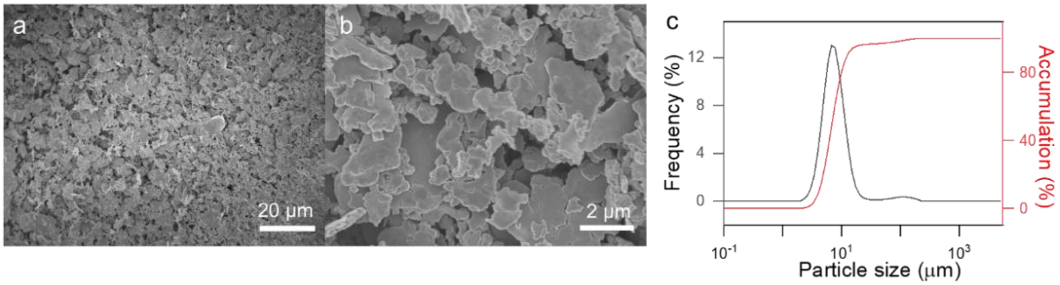

Supplementary Fig. 26 | Size characterization of Ag flakes. SEM (high-magnification (a) and low-magnification (b)) and particle size distribution (c) of used Ag flakes.

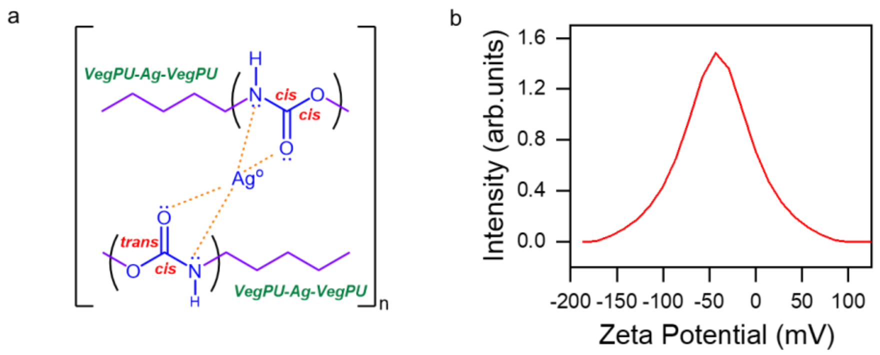

Supplementary Fig. 27 | The surface properties of Ag flakes. Schematics of the bonding between Ag flakes coated by fatty acid and VegPU (a) and the zeta potential of Ag flakes in the VegPU resin (b).

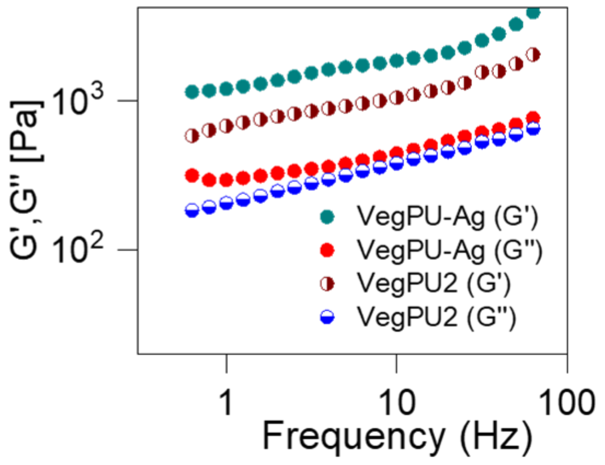

Supplementary Fig. 28 | The rheology of VegPU and VegPU/Ag. The dependence of the storage modulus (G') and lost modulus (G'') on angular frequency for VegPU resin and VegPU/Ag flakes ink.

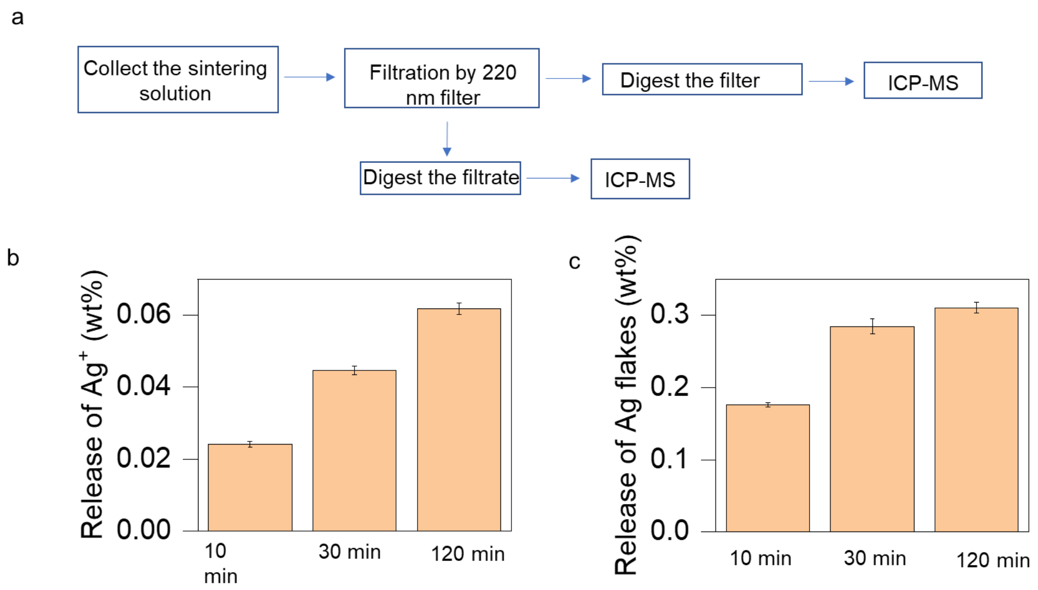

Supplementary Fig. 29 | Release of Ag flakes and Ag^{+} during the sintering solution curing process: (a) the process of collecting Ag flakes and Ag^{+}; The release of Ag^{+}(b) and Ag flakes (c) upon soaking duration. Error bar: n=3.

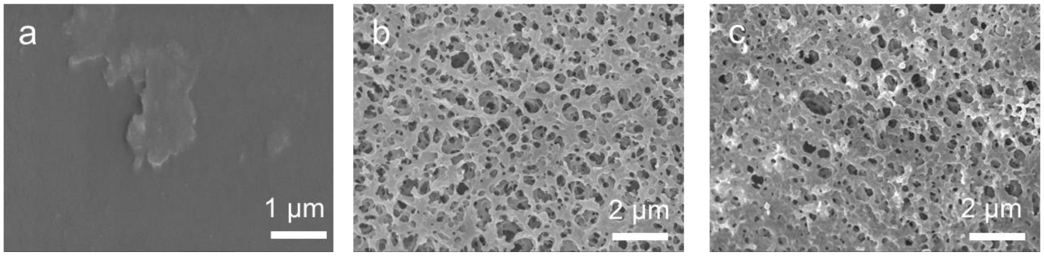

Supplementary Fig. 30 | The morphology of VegPU film treated by different methods. (a-c) The SEM images of VegPU resin after being cured by 80 °C heat, DI water, and sintering solution, respectively.

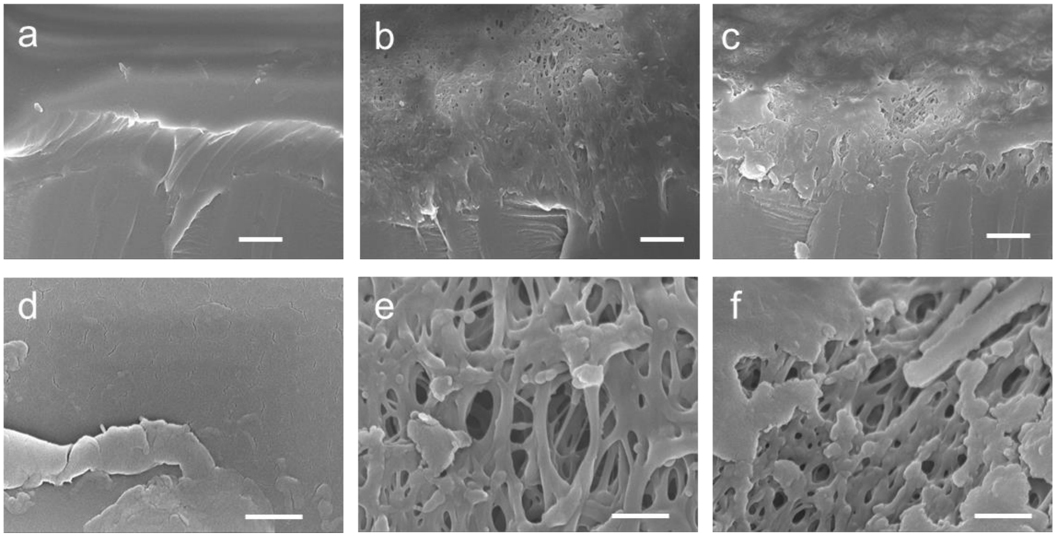

Supplementary Fig. 31 | Cross-sectional SEM images of VegPU treated by different methods. (a-c) The cross-sectional SEM images of VegPU resin after cured by 80 ℃ heat, DI water, and sintering solution, respectively. (d-f) The corresponding SEM images with a higher magnification. Scale bar in (a-c): 5 µm and in (d-f) 1 µm.

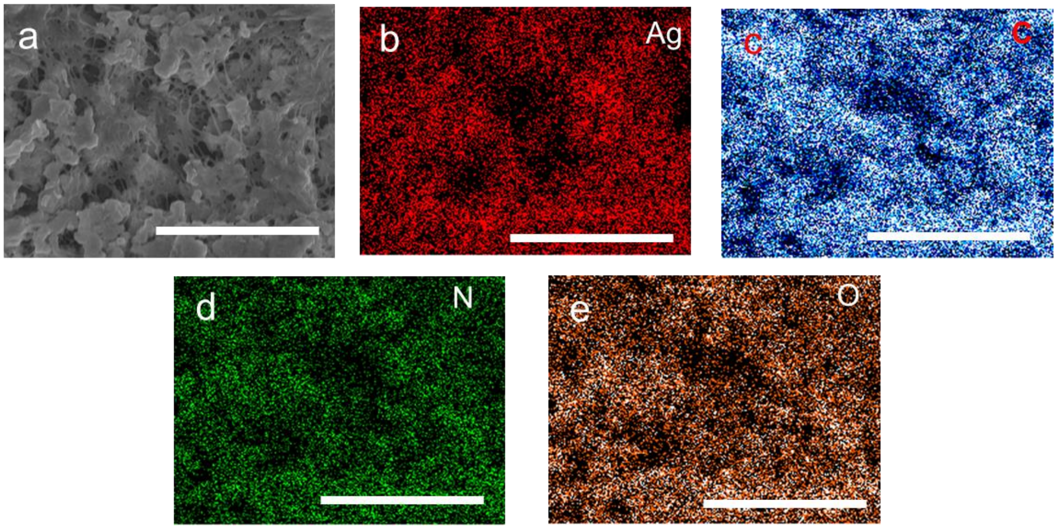

Supplementary Fig. 32 | EDX images of VegPU/Ag. (a) SEM image of the sintering solution-cured electrode and corresponding EDX images showing Ag (b), C (c), N (d), and O (e) elements. Scale bar: 6 µm.

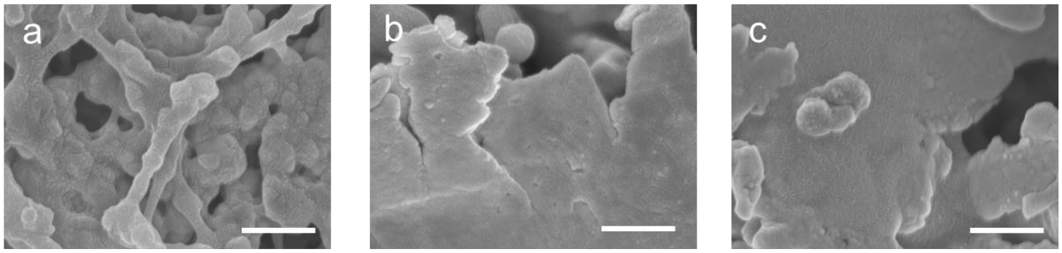

Supplementary Fig. 33 | The morphology of VegPU/Ag film treated by different methods. (a-c) The high-resolution SEM images of sintering solution-, DI water- and heat-cured VegPU/Ag electrodes, respectively. Scale bar: 500 nm.

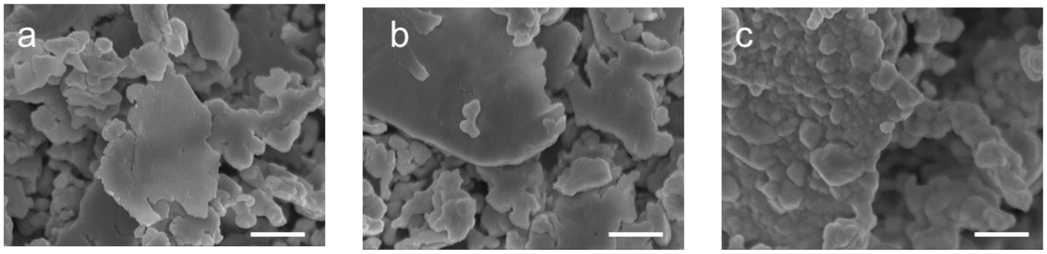

Supplementary Fig. 34 | The morphology of Ag flakes treated by different methods. (a-c) The SEM images of Ag flakes, DI water, and sintering solution-treated Ag flakes, respectively. Scale bar: 1 μm.

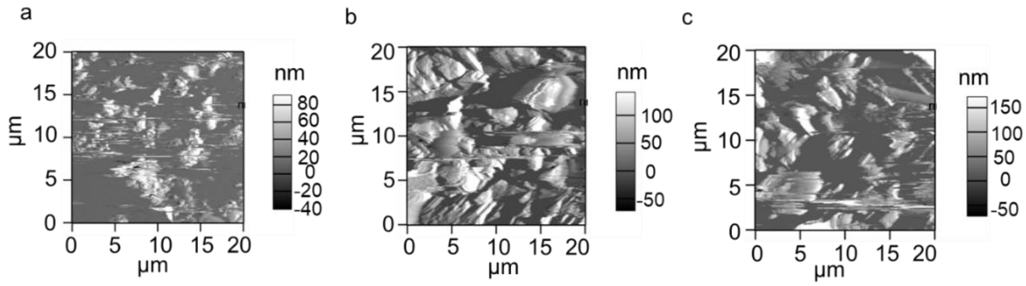

Supplementary Fig. 35 | C-AFM topography images of VegPU/Ag electrodes cured by heat curing (a) sintering solution (b) and water (c).

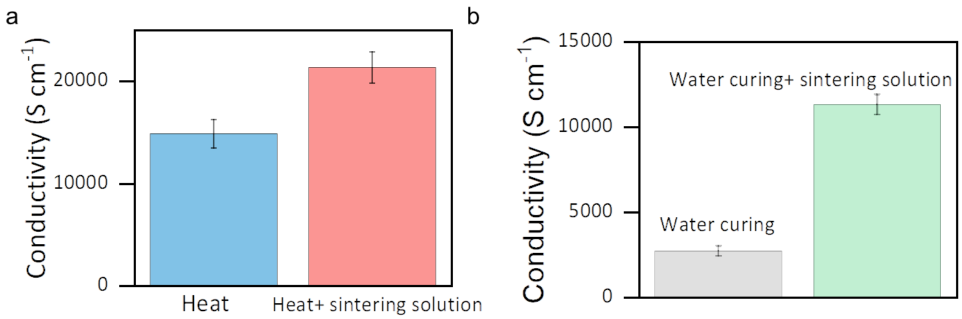

Supplementary Fig. 36 | The effect of sintering solution treatment on the conductivity of the electrodes cured by heat and water. The heat-treated (a) and DI water-treated (b) electrodes were retreated by the sintering solution. Error bar: n=3.

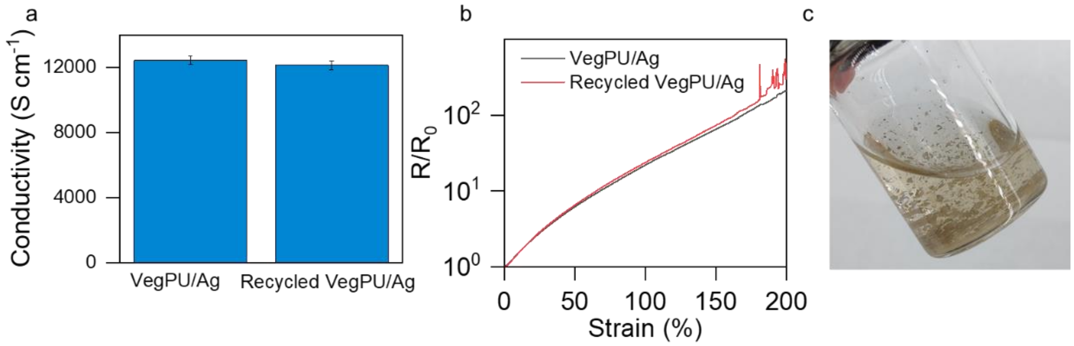

Supplementary Fig. 37 | The recycling of the VegPU/Ag electrode. The conductivity (a) and resistance changes upon stretching (b) of recycled VegPU-based conductor. (c) The photo image of collected Ag flakes dispersed in the DMF solvent. Error bar: n=3.

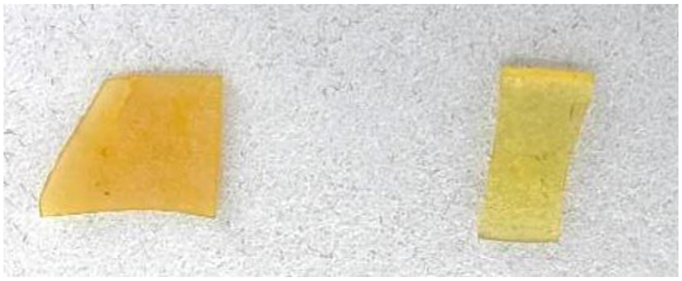

Supplementary Fig. 38 | The degradation of VegPU film. The photo images of VegPU after soaking in PBS buffer solutions with (left) and without (right) the presence of lipase enzyme.

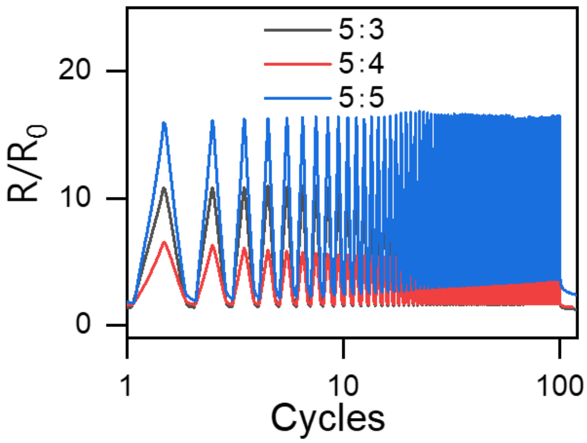

Supplementary Fig. 39 | The resistance changes of VegPU during cyclic stretching with different ratios of Ag and VegPU. The stretching rate is 60 mm/min. VegPU electrodes were stretched to 50% extension and then released at the same rate.

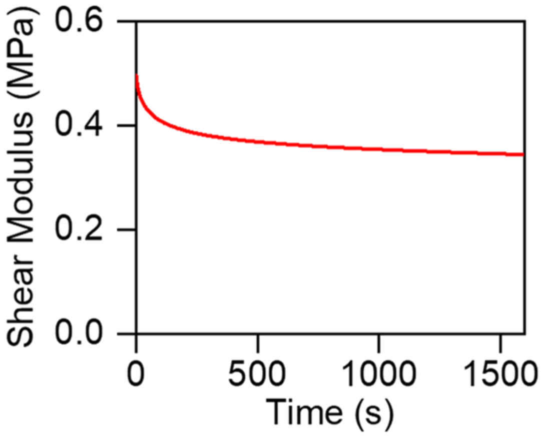

Supplementary Fig. 40 | The stress relaxation of VegPU film. The test was performed at the ambient temperature (20 °C).

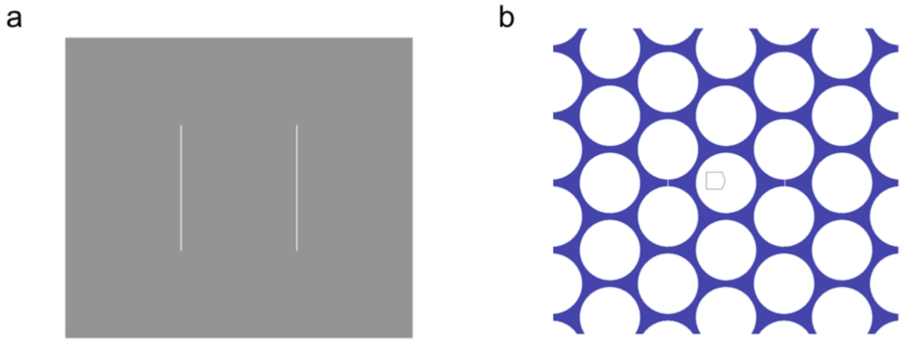

Supplementary Fig. 41 | The pre-cut cracks in the dense (a) and porous VegPU film (b). The cracks in two films with a same length.

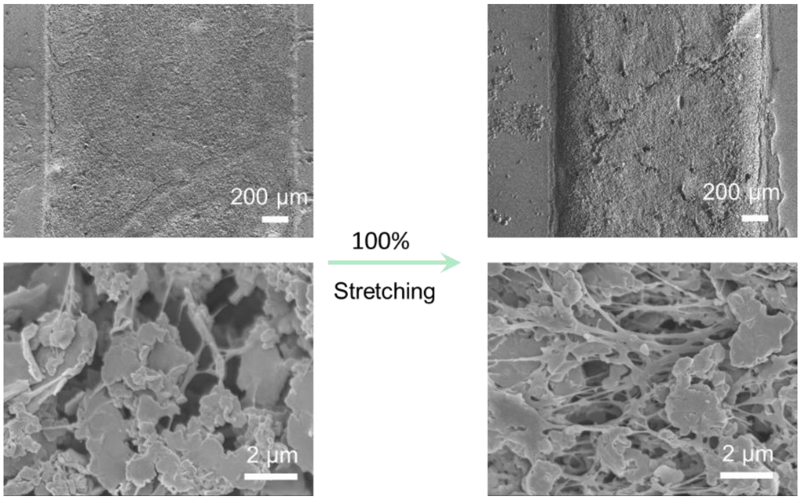

Supplementary Fig. 42 | SEM images of the water-treated electrode with the presence 100% of stretching. Under 100% strain, there are no macro cracks on the water-treated electrode.

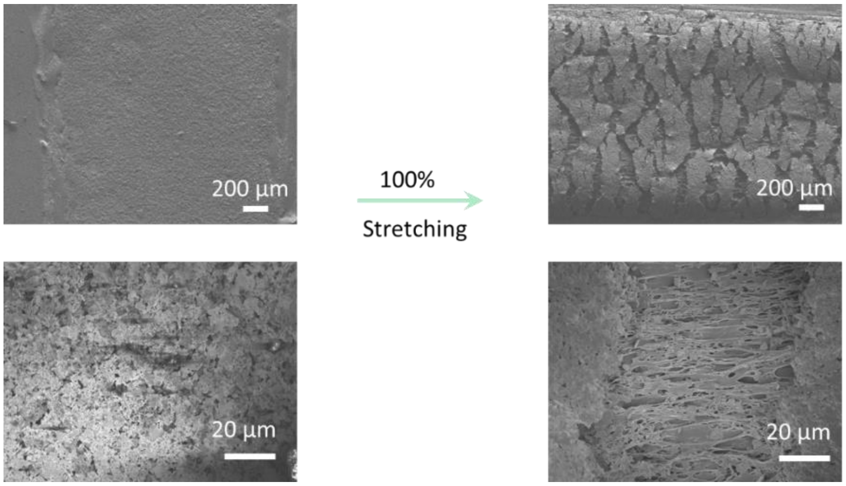

Supplementary Fig. 43 | SEM images of the heat-treated electrode with the presence of stretching. The stretching level is 100%. The printed electrode was adhered on a sample stage.

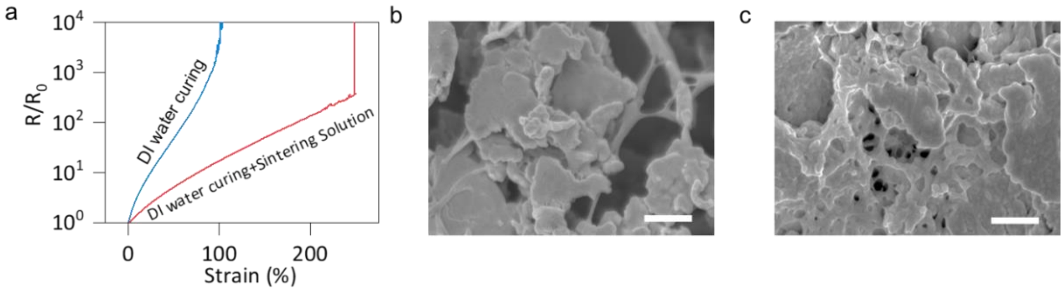

Supplementary Fig. 44 | The effect of sintering solution treatment on the DI water-cured electrode. The resistance variation upon strain (a) and SEM images of VegPU/Ag electrodes cured by DI water without (b) and with (c) subsequent sintering solution, scale bar: 1 µm.

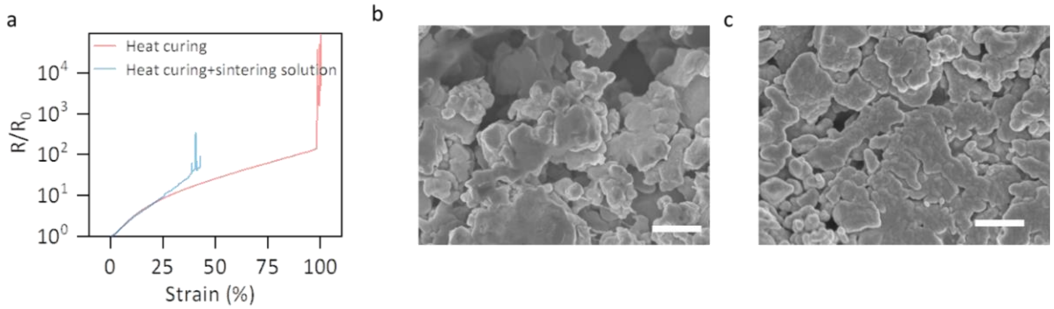

Supplementary Fig. 45 | The effect of sintering solution treatment on the heat-treated electrode. The strain-resistance change curve (a) and SEM images of the heat-cured VegPU/Ag electrode before (b) and after (c) the retreatment by sintering solution, scale bar: 1 µm.

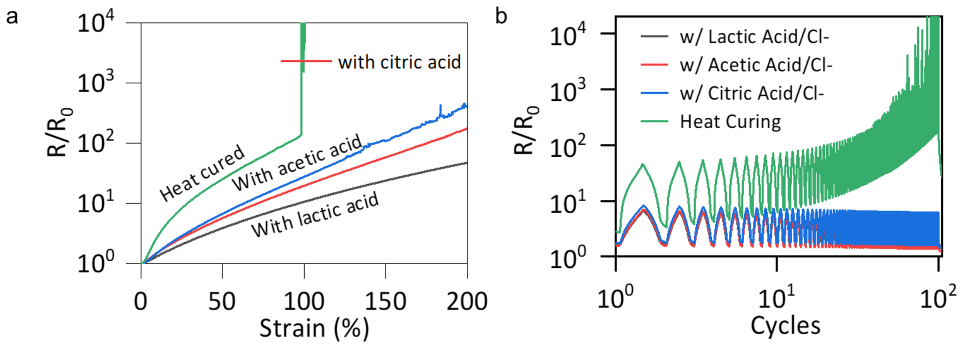

Supplementary Fig. 46 | The strain-resistance change (a) and cycling stretching (b) curves of VegPU/Ag electrodes cured by different sustainable sintering solutions (lactic acid/Cl^-, acetic acid/Cl^- and citric acid/Cl^-).

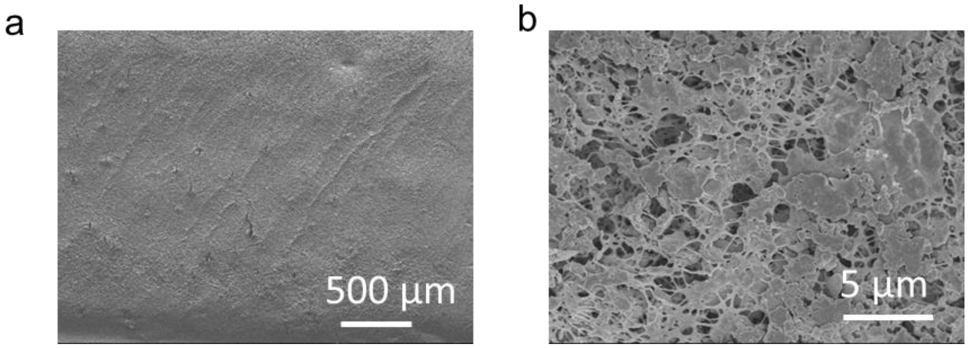

Supplementary Fig. 47 | The SEM images of sintering solution-treated electrode after 1000 cycles of 50% stretching: (a) Low magnification and (b) high magnification.

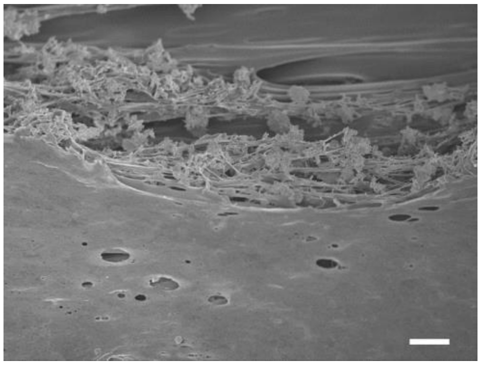

Supplementary Fig. 48 | The SEM images of encapsulated sintering solution-treated VegPU/Ag electrode. The encapsulation material was VegPU and the SEM image was taken under 50% strain, scale bar: 10 μm.

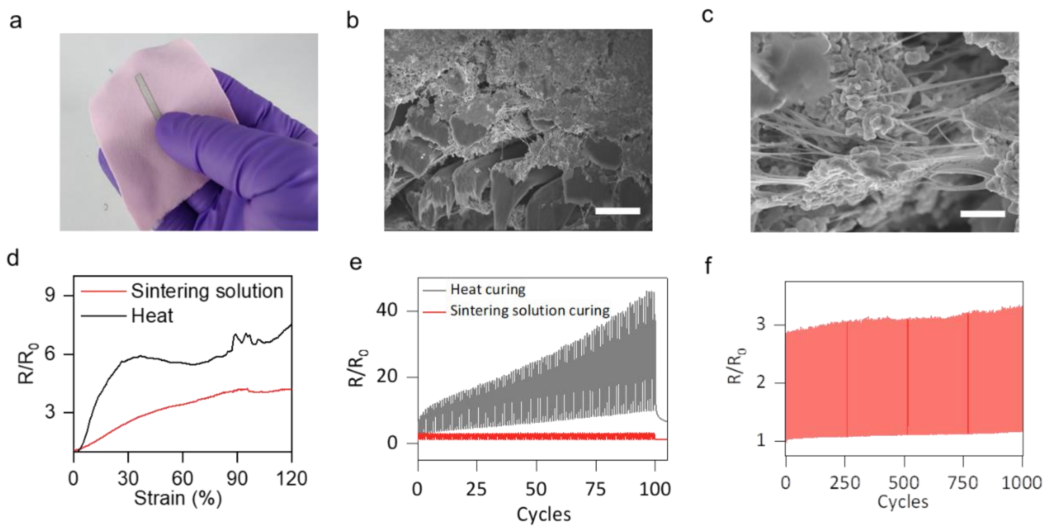

Supplementary Fig. 49 | The performance of VegPU/Ag printed on a textile substrate. The photo image (a), SEM images (b and c), strain-resistance change curve (d), and resistance change during 50% cycling for 100 cycles (e) and 1000 cycles (f) of the sustainable VegPU/Ag electrode printed on textile. Scale bar: 20 µm.

Supplementary Table 2 | The performance comparison of VegPU/Ag conductors with reported conductors.

Materials	structure	Process	Conductivity ($S\ cm^{-1}$)	Stretchability	Cyclic degradation	Test conditions	number
Ag flakes/ fluorine rubber	film	Screen printing	6168	400%	500	50% 100 cycles	8
Ag flakes/ Polyester	film	Screen printing	7500	170%	25	50% 100 cycles	9
Ag flakes/PDMS	film	Screen printing	10604	119%	50%<br>3.5	30% 1000 cycles<br>80% 1000 cycles	10
Ag flakes/TPU	Film	Direct writing	10000	240%	3.5	5% 1000 cycles	11
Ag flakes/PDMS-MPU-IU	film	Transfer printing	632	3500%	3	50% 1000	12
Ag flakes/ECO-flex	film	Screen printing	2400	500%	0.1	50% 1000 speed 4.4 mm/min	13
Ag flakes-Ag coated PDMS/PDMS	film	Stencil printing	1190	125%	90	50% 1000 cycles	14
Ag flakes/4-arm PEG/PANI/PTA	film	Stencil printing	10800 (4-8 order low before stretching)	1000%	n	1000% 1000 cycles	15
Ag flakes/PCL/Enzyme	film	3D printing	210	80%	0.875	15% 1000 cycles	16
VegPU/Ag flakes	film	Stencil printing	12833	350%	0.245<br>0.333	50% 1000 cycles<br>100% 1000 cycles	Our work
	Textile	Stencil printing		125% (limited by textile substrate)	0.182	50% 1000 cycles	

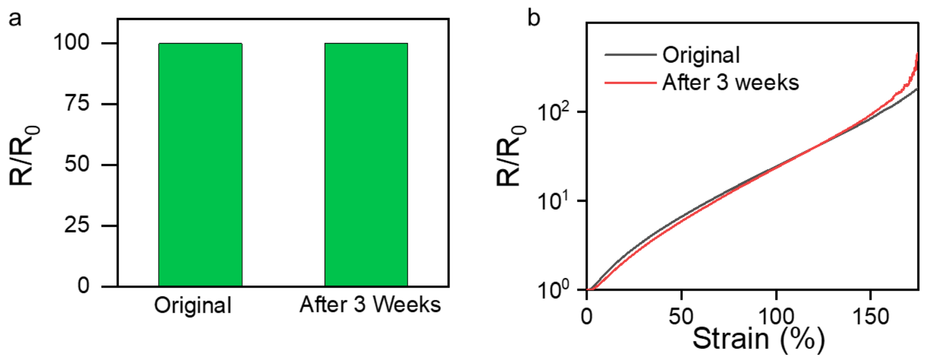

Supplementary Fig. 50 | The resistance (a) and stretchability (b) of VegPU/Ag after 3 weeks. The VegPU/Ag electrodes without any encapsulation was stored at ambient temperature (20 ℃).

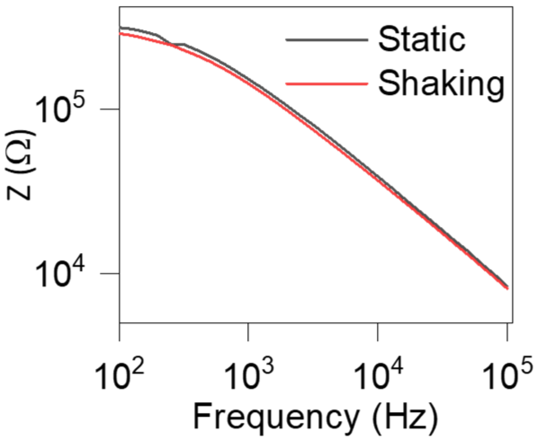

Supplementary Fig. 51 | Bode impedance curves of tomato during shaking. The voltage is 0.2 V with frequencies from 100 Hz to 100000 Hz.

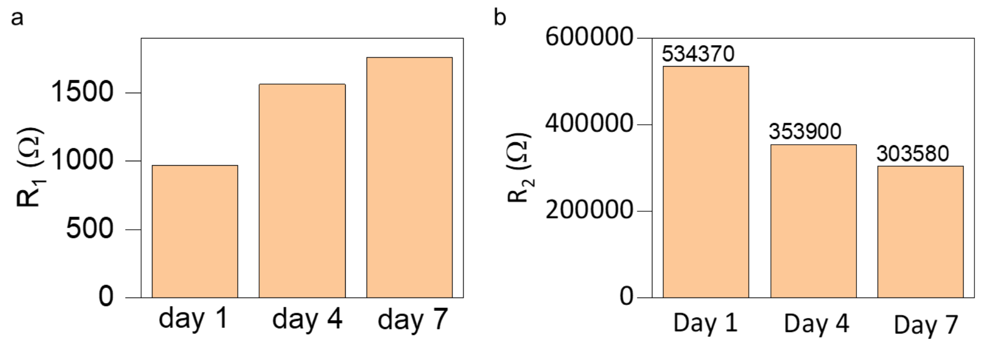

Supplementary Fig. 52 | The change of R_1 (a) and R_2 (b) during one-week storage. The intra- and extra-cellular fluids are modeled by R_1 and R_2. The tomato was stored at ambient temperature for 7 days.

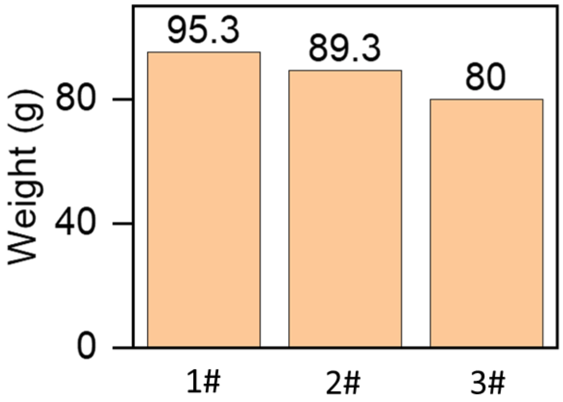

Supplementary Fig. 53 | The weight of three targeted tomatoes. The weight of three tomatoes was measured by a balance (ME 204, Mettler Toledo).

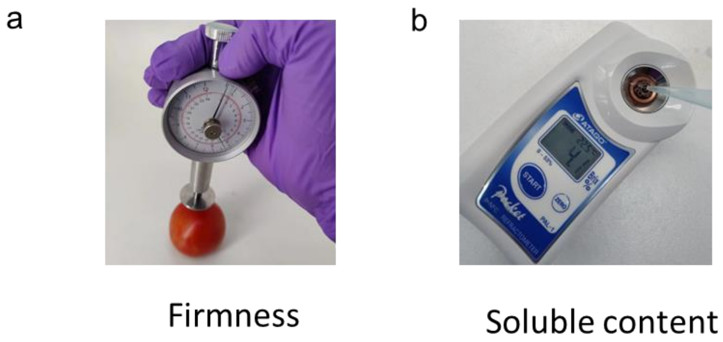

Supplementary Fig. 54 | The photo images of firmness (a) and soluble content test (b). The firmness of the selected tomato was tested by a fruit hardness tester (GY-3, Jingcheng Instrument), while the soluble content of the tomato juice was measured by a refractometer (PAL-1, ATAGO).

Supplementary References:

1. Ma, R. & Tsukruk, V. V. Seriography-Guided Reduction of Graphene Oxide Biopapers for Wearable Sensory Electronics. *Advanced Functional Materials* **27**, 1604802 (2017).
2. Lv, J. *et al.* Printable elastomeric electrodes with sweat-enhanced conductivity for wearables. *Science Advances* (2021) doi:10.1126/sciadv.abg8433.
3. Xiong, J., Thangavel, G., Wang, J., Zhou, X. & Lee, P. S. Self-healable sticky porous elastomer for gas-solid interacted power generation. *Sci. Adv.* **6**, eabb4246 (2020).
4. Parida, K. *et al.* Extremely stretchable and self-healing conductor based on thermoplastic elastomer for all-three-dimensional printed triboelectric nanogenerator. *Nat. Commun.* **10**, 2158 (2019).
5. Shen, Y. *et al.* Synthesis and characterization of vegetable oil based polyurethanes with tunable thermomechanical performance. *Industrial Crops and Products* **140**, 111711 (2019).
6. Alagi, P., Choi, Y. J., Seog, J. & Hong, S. C. Efficient and quantitative chemical transformation of vegetable oils to polyols through a thiol-ene reaction for thermoplastic polyurethanes. *Industrial Crops and Products* **87**, 78–88 (2016).
7. Zeng, Y. *et al.* Highly Stretchable Fatty Acid Chain-Dangled Thermoplastic Polyurethane Elastomers Enabled by H-Bonds and Molecular Chain Entanglements. *ACS Sustainable Chem. Eng.* **10**, 11524–11532 (2022).
8. Matsuhisa, N. *et al.* Printable elastic conductors by in situ formation of silver nanoparticles from silver flakes. *Nat. Mater.* **16**, 834–840 (2017).
9. Nam, H. J., Kang, S. Y., Park, J. Y. & Choa, S.-H. Intense pulse light sintering of an Ag microparticle-based, highly stretchable, and conductive electrode. *Microelectronic Engineering* **215**, 111012 (2019).

10. Guo, W. *et al.* Matrix-Independent Highly Conductive Composites for Electrodes and Interconnects in Stretchable Electronics. *ACS Appl. Mater. Interfaces* **11**, 8567–8575 (2019).
11. Valentine, A. D. *et al.* Hybrid 3D Printing of Soft Electronics. *Advanced Materials* **29**, 1703817 (2017).
12. Kim, S. H. *et al.* An Ultrastretchable and Self-Healable Nanocomposite Conductor Enabled by Autonomously Percolative Electrical Pathways. *ACS Nano* **13**, 6531–6539 (2019).
13. Oh, Y. *et al.* Selective photonic sintering of Ag flakes embedded in silicone elastomers to fabricate stretchable conductors. *J. Mater. Chem. C* **5**, 11733–11740 (2017).
14. Pan, C. *et al.* Silver-Coated Poly(dimethylsiloxane) Beads for Soft, Stretchable, and Thermally Stable Conductive Elastomer Composites. *ACS Appl. Mater. Interfaces* **11**, 42561–42570 (2019).
15. Wang, T., Liu, Q., Liu, H., Xu, B. & Xu, H. Printable and Highly Stretchable Viscoelastic Conductors with Kinematically Reconstructed Conductive Pathways. *Advanced Materials* **34**, 2202418 (2022).
16. Kwon, J. *et al.* Conductive Ink with Circular Life Cycle for Printed Electronics. *Advanced Materials* **34**, 2202177 (2022).
