# Supplementary material for: Printed sustainable elastomeric conductor for soft electronics
Source: Nat Commun. 2023 Nov 6;14:7132. doi: 10.1038/s41467-023-42838-7 (PMC10628110; doi:10.1038/s41467-023-42838-7)
Supplement: Supplementary file 4 — Description of Additional Supplementary Files [file 41467_2023_42838_MOESM4_ESM.pdf]

Description of additional supplementary files

Supplementary Movie 1 | The demonstration of sintering solution treatment.

Supplementary Movie 2 | Tomato impedance sensing by a soft gripper.
